# Interactions between functionalised silica nanoparticles and *Pseudomonas fluorescens* biofilm matrix: A focus on the protein corona

**DOI:** 10.1371/journal.pone.0236441

**Published:** 2020-07-23

**Authors:** Caio H. N. Barros, Stephanie Fulaz, Stefania Vitale, Eoin Casey, Laura Quinn

**Affiliations:** School of Chemical and Bioprocess Engineering, University College Dublin, Dublin, Ireland; Luleå University of Technology, SWEDEN

## Abstract

Biofilms are microbial communities embedded in an extracellular polymeric matrix and display an enhanced tolerance to the action of antimicrobials. The emergence of novel functionalised nanoparticles is considered a promising avenue for the development of biofilm-specific antimicrobial technologies. However, there is a gap in the understanding of interactions between nanoparticles and the biofilm matrix. Particularly, questions are raised on how nanoparticle charge and surface groups play a role in aggregation when in contact with biofilm components. Herein we present the synthesis of four types of silica nanoparticles and undertake an analysis of their interactions with *Pseudomonas fluorescens* biofilm matrix. The effect of the biofilm matrix components on the charge and aggregation of the nanoparticles was assessed. Additionally, the study focused on the role of matrix proteins, with the in-depth characterisation of the protein corona of each nanoparticle by Liquid Chromatography with Tandem Mass Spectrometry experiments. The protein corona composition is dependent on the nanoparticle type; non-functionalised nanoparticles show less protein selectivity, whereas carboxylate-functionalised nanoparticles prefer proteins with a higher isoelectric point. These outcomes provide insights into the field of biofilm-nanoparticle interactions that can be valuable for the design of new nano-based targeting systems in future anti-biofilm applications.

## Introduction

Although bacteria are traditionally studied in their planktonic state, the biofilm phenotype is more preeminent and a highly successful mode of life on Earth [1, 2]. A biofilm is a homogeneous or heterogeneous aggregation of microorganisms in which the bacterial cells are encased in a self-produced matrix made up of Extracellular Polymeric Substances (EPS) [3]. In recent decades, the in-depth study of biofilms has highlighted the critical role bacterial adhesion to a surface has for biofilm development [4].

The biofilm matrix is comprised of proteins, carbohydrates and extracellular DNA (eDNA) [5]. Each EPS component has a distinct orchestrated function in the microbial community, such as adhesion (polysaccharides, proteins and eDNA), cohesion (amyloids, lectins), retention of water (hydrophilic polysaccharides), sorption of inorganic and organic compounds, among other roles [6]. The matrix is an important feature of biofilms that differentiates them from the planktonic phenotype, being responsible not only for the overall architecture but also for the physical and biochemical defence against antimicrobials [7]. In fact, the intensified drug resistance of several microbial strains is in part due to the existence of the EPS matrix [8]. Another complicating factor is that the EPS composition is highly dependent on the strain that produces it; as a result, it is somewhat difficult to study and propose eradication methods that can be effective throughout diverse strains.

Due to the low susceptibility to common antibiotics and anti-biofouling agents [9] and especially because of their hindered penetration and inactivation into the matrix of biofilms, NPs have recently been used as biofilm probes [10], antimicrobial nanocarriers [11–13] and/or biofilm dispersion agents themselves [14]. Factors such as size, charge and surface chemistry can be tuned to achieve NP adsorption and penetration into the EPS matrix [15, 16]. However, there is a discrepancy between the number of studies performed to eradicate biofilms when compared to the number of studies that look solely at interactions between NPs and the biofilm matrix [17]. A more fundamental understanding of the interactions between NP surface and EPS components is still lacking in order to provide a solid basis for the design of smart NPs with anti-biofouling properties that can effectively penetrate into the biofilm.

This study is based on the premise that NP surface functionalisation may affect biomolecule (especially protein) corona formation [18], which can in turn influence mechanisms that are involved in nano-based antibiofilm processes such as NP aggregation, bacterial adhesion, and NP uptake. Here, *Pseudomonas fluorescens* biofilms were grown at the air-liquid interface and had their EPS extracted using a cation exchange resin to weaken the overall matrix architecture, which is maintained by Ca^2+^ and Mg^2+^ bridges [19, 20]. Functionalised silica NPs were then used for *in vitro* assays with the extracted EPS to evaluate how the matrix components affect charge and aggregation of NPs. As these biofilms are protein-rich, a special focus was given to the identification of proteins which form a corona around the NPs and how surface chemistry tunes the protein corona composition. Therefore, the aim of this study centred on evaluating how the surface chemistry of a NP can yield an impact on its aggregation behaviour as well as on the identity of the protein corona formed when in contact with isolated biofilm matrix. Thus, the information gathered here provides crucial evidence that aids in the rational design of smart NPs with tuneable surface properties for specific purposes based on EPS-NP interactions.

## Materials and methods

### Materials

King B Agar, peptone (vegetable) No.1, tetracycline hydrochloride, gentamicin sulphate salt, calcium chloride (CaCl_2_), magnesium sulphate (MgSO_4_), acetic acid, ammonium chloride (NH_4_Cl), ammonium hydroxide (NH_4_OH), absolute ethanol (EtOH), tetraethyl orthosilicate (TEOS), (3-aminopropyl) triethoxysilane (APTES), N1-(3-trimethoxysilylpropyl) diethylenetriamine (DETA), dimethylformamide (DMF), N-hydroxysuccinimide (NHS), 1-ethyl-3-(3-dimethylaminopropyl)carbodiimide (EDC), dithiothreitol (DTT), iodoacetic acid, acetic acid, acetonitrile, formic acid, sodium alginate, bovine serum albumin protein standard (BSA), Dowex^®^ cation exchange resin, glycerol and trypsin (MS grade), 4,5,6,7-tetrachloro-2’,4’,5’,7’-tetraiodofluorescein disodium salt (Rose Bengal) and ninhydrin were purchased from Sigma Aldrich and utilised as supplied. Potassium phosphate dibasic was purchased from Honeywell, Fluka^™^. The water used in all experiments was MilliQ grade water, purified using an Elga Process Water System.

### Nanoparticle synthesis

Bare silica NPs (silica NPs) were synthesised in an adapted Stöber sol-gel method [21]. Briefly, 1.43 mL of TEOS was added to 20 mL of methanol at 40 °C. Then, 2.2 mL of NH_4_OH was added and the reaction was carried out for 1 h, under constant stirring. The NPs were washed by centrifugation (3 times, 9,000 rpm, 15 min). For insertion of amine groups onto the NPs, the silane DETA was used. In this reaction, 60 mg of silica NPs were dissolved in 23.0 mL of acetic acid 1 mmol L^-1^ and DETA (100 μL in 10.0 mL acetic acid 1 mmol L^-1^) was added to the suspension. The reaction was carried out for 30 min at room temperature under constant stirring and washed as described above to obtain amine-functionalised silica NPs (amine-NPs). Two further functionalisation reactions were then performed: insertion of a carboxylate group (—COOH functionalised silica NPs–carboxylate-NPs) and insertion of an aromatic group (aromatic- functionalised silica NPs—aromatic-NPs). To obtain the former, 10 mg of amine-NPs were suspended in 6 mL of DMF followed by addition of a solution of succinic anhydride (440 mg) in 6 mL of DMF. After stirring at room temperature for 3 days, carboxylate-NPs were recovered by washing firstly with DMF, and then two times with deionised water. Aromatic-NPs were synthesised by activating benzoic acid (96 mg) in 20 mL of deionised water with the addition of NHS (288 mg in 2 mL deionised water) and EDC (300 mg in 2 mL deionised water) for 2 h at 30 °C with constant stirring. Then, 35 mg of amine-NPs suspended in 9 mL of deionised water were added and the reaction proceeded overnight under stirring at room temperature. Aromatic- functionalised silica NPs were obtained after a number of washing steps with water and ethanol.

### Nanoparticle characterisation

Dynamic Light Scattering (DLS) and Zeta potential measurements were performed in a Zetasizer Nano ZS (Malvern Instruments) in the facilities of the Centre for BioNano Interactions (CNBI). NPs samples in 1 mg mL^-1^ aqueous dispersion were analysed in a folded capillary zeta cell. For size measurements, experiments were run in duplicate or triplicate, with 10 runs for each measurement and duration of 10 s for each scan. For zeta potential measurements, all experiments were done in triplicate, with 10 scans each. Scanning Electron Microscopy (SEM) imaging was carried out using an SEM FEI Quanta 3D FEG Dual Beam SEM, with silica NPs in powder form being deposited on carbon conductive adhesive tapes. At least 3 images and 100 nanoparticles per sample were considered to estimate NPs size. FTIR was performed in a Bruker Vertex 70 spectrophotometer. Samples in powder form were adhered onto NaCl FTIR cards using a dispersion in ethanol and drying it at 70 °C. Spectra were obtained using a resolution of 4 cm^-1^, spectral window from 4000 to 400 cm^-1^ and 64 scans. Dissolution NMR was used to analyse the NPs surface groups, based on a previously reported method [22]. Silica NPs in the powder form (5–10 mg) were suspended in 662 μL of D_2_O and then 38 μL of NaOD was added. The mixture was incubated at 37 °C overnight or until a clear solution was obtained. ^1^H NMR spectra were then obtained in a Varian Inova 300 MHz Spectrometer using 128 scans per sample.

The density of amino groups at the NP surface after the functionalisation steps was estimated via the ninhydrin assay. A silica NPs sample (1.5–6 mg) was added to 1 mL of ninhydrin ethanolic solution (0.175 mol L^-1^). After sonication for 40 min, the mixture was left in a water bath (70–90 °C) in 2 mL tubes for 25 min. The nanoparticles were then collected via centrifugation (9,000 rpm, 10 min) and the absorbance of the supernatant was read at 570 nm. A calibration curve was built using solutions of the silane DETA at various concentrations.

Estimation of surface hydrophobicity was done using the Rose-Bengal assay. A volume of 100 μL of Rose Bengal dye at 200 μg mL^-1^ was added to 900 μL of NPs aqueous suspensions of various concentrations followed by incubation for 3 h at 25 °C. The NPs were isolated via centrifugation (12,000 rpm, 3 min) and the absorbances of the supernatants were read at 549 nm for determination of free dye in solution. In order to estimate the hydrophobicity of a NP sample, a plot of the partitioning quotient (amount of bound dye divided by amount of free dye) versus total surface area of NPs was made.

### Biofilm cultivation and EPS extraction

Using a glycerol stock, mCherry-expressing *P*. *fluorescens* (PCL 1701) was streaked onto a King B agar plate containing gentamycin (10 μg mL^-1^) and incubated at 30 °C for 24 h. A single colony was used to inoculate a sterile conical flask containing 50 mL of King B media and 50 μL of gentamicin (10 μg mL^-1^). The culture was incubated for 16–18 h at 30 °C, 200 rpm. The OD_600_ of the overnight culture was adjusted to 1 using sterile King B media. The bacterial culture was supplemented with CaCl_2_ (1.5 mM) and gentamicin (10 μg mL^-1^) and 5 ml of the bacterial mixture was added to a falcon tube containing a previously sterilised coverslip. The tubes were incubated at 30 °C for 72 h at 100 rpm.

For the extraction of the EPS, the procedure was adapted from the one described by Wu and Xi (2009) [23]. The coverslips containing biofilms were first washed with PBS buffer and then resuspended after scraping in 0.5 mL of NaCl 0.9% (per tube). After sonication for 2 minutes, 1% Dowex^®^ resin (50 mg mL^-1^) was added. The tubes were incubated at 4 °C for 2 h at 300 rpm in the dark and then centrifuged at 4 °C for 30 min at 13,500 rpm. The supernatant containing the EPS matrix was taken off and filtered through a 0.22 μm pore membrane. The EPS suspension was stored at -20 °C overnight or at -80 °C for up to a month.

### EPS characterisation

Proteins and sugars contained in the extracted EPS solution were quantified. The proteins in the extracted EPS were quantified using the Lowry assay [24] by preparing a calibration curve with BSA as a standard. For the quantification of sugars, the colorimetric Phenol-Sulfuric acid method [25] was used. All measurements were made in triplicate in a plate reader (SpectraMax iD3, Molecular Devices).

### Zeta potential and DLS of silica NPs-EPS solutions

NPs were dispersed at a concentration of 1 mg mL^-1^ in solutions of EPS concentrations ranging from 200 to 6.25 μg mL^-1^. After one hour in a sonication bath they were analysed by DLS and Zeta potential measurements, as described previously. In order to isolate the contributions of polysaccharides and proteins, solutions of alginate (3.50 to 0.05 μg mL^-1^) and BSA (8.00 to 0.25 μg mL^-1^) were used to disperse the NPs again and analyse them through the same method.

### Gel electrophoresis

Using a Bio-Rad PowerPac^™^ and gel cassette, the total proteins of the extracted EPS and protein corona of a given silica NPs sample were run through a denaturing polyacrylamide gel (SDS-PAGE 12%). In all cases, 10 μL of the sample was mixed with 10 μL of sample buffer (1.25 mL of 1 M Tris-HCl (pH 6.8), 4.0 mL 10% (w/v) SDS, 2.0 mL glycerol, 0.5 mL of 0.5 M EDTA, 4 mg bromophenol blue, 0.2 mL β-mercaptoethanol, 2.05 mL distilled H_2_O), incubated at 90 °C for 5 min and inserted into the wells of the stacking gel. After immersion of the gel cassette into a running buffer (3.03 g Tris-base, 14.4 g glycine, 1.0 g SDS in 1 L of distilled water), an electric potential of 120 V was applied for the first 20 min and then 150 V until the run was finished. For analysis of the protein corona, the procedure was adapted from the literature [26]. To 100 μL of a suspension of the NP (10 mg mL^-1^), 900 μL of extracted EPS was added. The mixture was incubated at 30 °C for 1 h and then centrifuged at 12,000 rpm for 20 min at 4 °C. The pellet was re-dispersed in 1 mL of water and again centrifuged. After removal of the supernatant, 10–30 μL of water was added to the pellet to generate a protein corona sample on the NPs.

### Protein corona identification by LC-MS/MS

To 100 μL of silica NPs at a concentration of 1 mg mL^-1^, 900 μL of extracted EPS was added, followed by incubation at 30 °C for 1 h at 800 rpm. The NPs were then washed twice (12,000 rpm, 4 °C, 20 min) via centrifugation and resuspended in 150 μL of water. The protein digestion protocol started by using 50 μL of EPS, to which 2.5 μL of DTT 100 mM were added followed by incubation for 30 min at 60 °C. Then, 2.6 μL of iodoacetic acid 200 mM was added, the mixture was vortexed and incubation was carried out in the dark at room temperature for 30 min. Ammonium bicarbonate buffer (50 mM pH 8) was added to a total volume of 0.5 mL and then 1.5 μL of trypsin solution (20 μg in 20 μL of acetic acid 50 mM) was added. Tryptic digestion took place during incubation overnight at 37 °C and was interrupted by addition of 5 μL of acetic acid 100%. For sample clean up, the supernatant containing fragmented proteins was isolated via centrifugation and then Solid Phase Extraction C_18_ cartridges (SepPak, Waters) were used for peptide isolation. For column conditioning, 1 mL of acetonitrile 0.1% formic acid was flushed through the cartridge, followed by 1 mL of deionised water 0.1% formic acid. The sample was slowly inserted in the cartridge and after complete injection, de-salinisation was carried out by flowing 1 mL of deionised water 0.1% formic acid. Finally, the peptides were collected by elution with acetonitrile 0.1% formic acid. The resulting solution was dried out under vacuum and resuspended in 30 μL of MS grade water containing 2.5% acetonitrile and 0.5% acetic acid. Peptides were analysed at UCD Conway Institute of Biomolecular and Biomedical Research using a quadrupole Orbitrap (Q-Exactive, Thermo Scientific) mass spectrometer equipped with a reversed-phase NanoLC UltiMate 3000 HPLC system (Thermo Scientific). Samples containing the peptides which were obtained from tryptic digestion were loaded onto C18 reversed phase columns (10 cm length, 75 μm inner diameter) using an injection volume of 5 μL and eluted with a linear gradient from 2 to 95% acetonitrile containing 0.5% acetic acid in 60 min at a flow rate of 250 nL min^-1^. The mass spectrometer was operated in data dependent mode, with automatic switch between MS and MS2 acquisition. A resolution of 70,000 was used and survey full scan MS spectra (m/z 350–1600) were acquired. MS2 spectra had a resolution of 17,500. The twelve most intense ions were sequentially isolated and fragmented by higher-energy C-trap dissociation.

MaxQuant software package was used for protein identification using the proteome database of *Pseudomonas fluorescens* F113 (extracted from Uniprot). Only proteins with a minimum number of peptides of 2 and minimum number of unique peptides of 1 were considered. Label-free quantification of the proteins was also run using the same software, in triplicate. Only proteins identified in all three replicates were considered. For statistical analysis, the Perseus framework was used.

## Results and discussion

### Nanoparticle synthesis and characterisation

**Fig 1:**
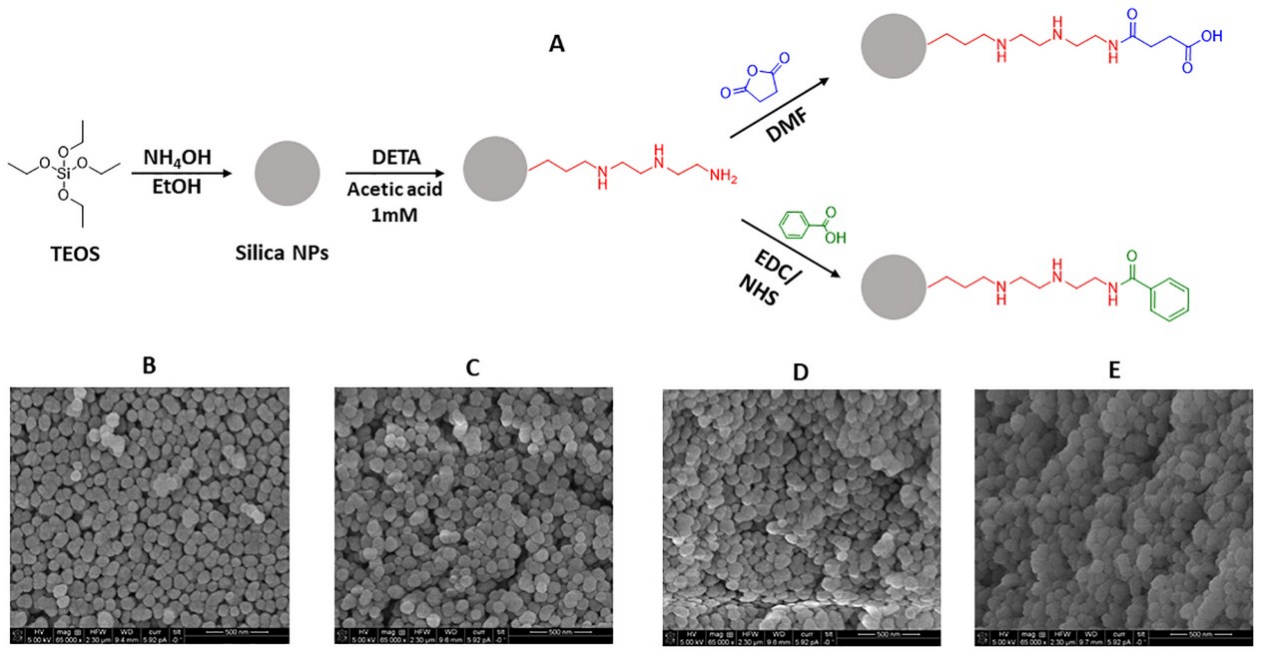
General scheme of synthesis and functionalisation of silica nanoparticles (A); Scanning Electron Microscopy (SEM) images of bare (B), amine-functionalised (C), carboxylate-functionalised (D) aromatic-functionalised (E) silica NPs.

The synthesis of silica NPs was optimised to yield NPs with approximately 100 nm in diameter. Following the synthesis of silica NPs, functional groups were inserted on their surface to provide distinct surface chemistries which are expected to lead to specific interactions inside the biofilm, facilitating penetration and/or co-localisation with specific components (proteins, sugars, etc.). The first functionalisation step chosen was the insertion of an amine group (using the silane DETA), which gives a positive charge to the NPs. From this positively charged NP, two further routes were taken: the insertion of a carboxylate group (using succinic anhydride as reagent and providing again a negative charge to the surface) or an aromatic group (using benzoic acid and maintaining a lower positive surface charge). Fig 1 depicts a scheme of the synthesis and functionalisation of the silica NPs and SEM images of the NPs, showing the preservation of a spherical and homogeneous morphology.

As seen in S1 Table in S1 Appendix, the hydrodynamic diameter of the NPs can be up to 60–70 nm larger than the actual core size seen in the micrographs. All NPs have a strong electrostatic stabilisation denoted by a high absolute value of zeta potential with the exception of the aromatic-NPs, which has a more neutral characteristic that can be ascribed to the insertion of an aromatic group. Nevertheless, all four types do not show any form of aggregation or precipitation in suspension for at least 2–3 months and are completely monodispersed, as shown in S1 Fig in S1 Appendix. The bare and carboxylate-NPs are considerably more stable than the other two (positively charged) types, being stable for up to a year after synthesis with no signs of aggregation. In order to confirm the presence of the surface chemical groups and, consequently, the success of the functionalisation reactions, FTIR spectra were obtained (S2 Fig in S1 Appendix). Some diagnostic absorption bands for silica NPs were observed in all samples [26] such as Si-O-Si symmetrical (830–841 cm^-1^) and asymmetrical (1100–1120 cm^-1^) stretching and Si-OH stretching (980–990 cm^-1^) vibration modes. The insertion of the amine-containing silane led to the appearance of alkane stretching bands (2940–2970 cm^-1^), which was preserved in the subsequent functionalizing steps. Additionally, the formation of amide bonds from the terminal amine (with either benzoic acid or succinic anhydride) introduced carbonyl stretching modes for the carboxylate and aromatic-NPs. A complete assignment of absorption bands is shown in S2 Table in S1 Appendix.

Dissolution ^1^H NMR was used to further confirm the surface composition after functionalisation. In this technique the problems that the silica suspension would cause to the analysis are eliminated by dissolving the NPs and maintaining only the surface groups in solution. As seen in S3–S6 Figs in S1 Appendix, the peaks related to the amine functionalisation (in red in S6 Fig in S1 Appendix) can be assigned to all the protons in the molecule. These peaks are maintained in the following functionalisation steps, with the addition of peaks assigned to α-carbonyl protons (blue) or aromatic protons (green). Therefore, the results obtained from zeta potential measurements, FTIR and NMR spectroscopies confirm the surface functionalisation of the NPs. The ninhydrin colorimetric assay was used to detect amine groups on the surface of the nanoparticle as a means to estimate the surface coverage of some of the functionalisation groups. After functionalisation with the silane DETA, it was calculated to be a density of 1.62 amine groups per nm^2^. This ratio decreased to 0.95 after insertion of the carboxylate moiety. The fact that there still was detection of amine groups for carboxylate-NPs may be explained by the presence of secondary amines in the DETA silane, which can also react with ninhydrin (S3 Table in S1 Appendix).

### EPS-nanoparticle interactions

The biofilm EPS was extracted using a cation exchange resin (CER), which exchanges cations (in this case, Ca^2+^ and Mg^2+^) thus weakening the bonds that maintain the matrix architecture. Proteins and carbohydrates are the major and most abundant biomolecules encased into the biofilm matrix [27, 28] therefore these two components were quantified in the extracted EPS using, respectively, the Lowry’s and Phenol-Sulfuric acid assays. The results are shown in S4 Table in S1 Appendix.

**Fig 2:**
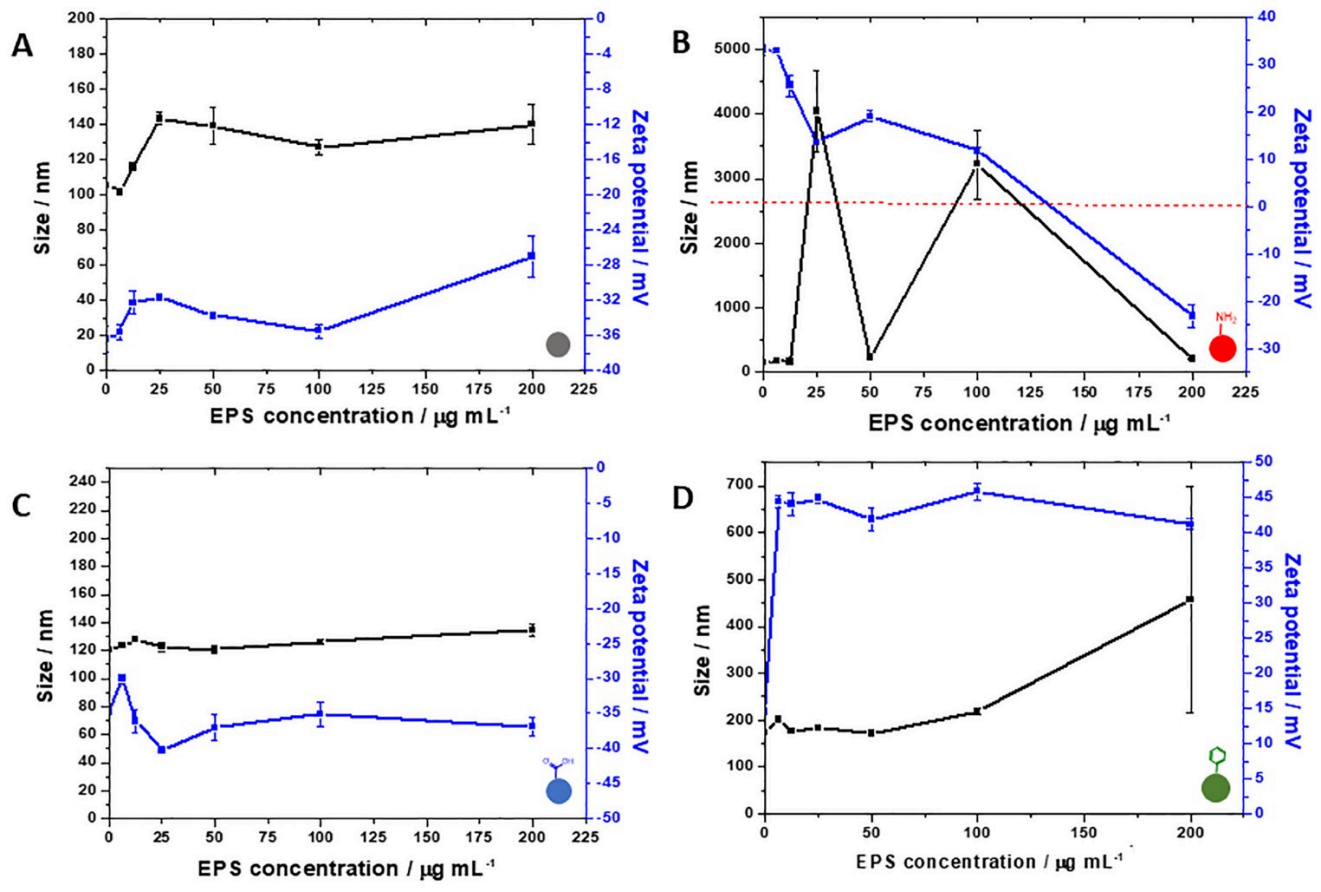
Influence of increasing concentrations of extracted EPS on zeta potential (■) and size (■) of bare (A), amine-functionalised (B), carboxylate-functionalised (C) and aromatic-functionalised (D) silica NPs.

The EPS extract was examined under light microscopy before the last centrifugation step in order to check cell lysis. No signs of lysed cells were seen, which indicates that all the EPS content is derived from the matrix and dead cells. In order to assess how the synthesised NPs would behave in the presence of the EPS matrix in biofilms, the silica NPs were exposed to various concentrations of extracted EPS in an attempt to mimic the conditions found inside the biofilm matrix. The hydrodynamic size and zeta potential values were recorded at each concentration (Fig 2).

These *in vitro* stability studies can give valuable insights on how NPs would behave inside a real biofilm. For instance, the effect of EPS on bare NPs is completely different from the one on amine-NPs; while the charge and size undergo slight changes for the former with increasing EPS concentrations, there is an inversion of charge (from positive to negative) for the latter with the formation of large aggregates as the zeta potential approaches neutrality. This effect is a clear demonstration of the Derjaguin-Landau-Verwey-Overbeek (DLVO) theory, as the repulsive electrostatic energy barrier that holds the colloid in suspension is overcome by the attractive van der Waals potential, leading to aggregation. This observation is in line with previously reported data from our group, in which confocal images of *P*. *fluorescens* interfacial biofilms exposed to amine-NPs revealed a high degree of aggregation onto the top layers of the biofilm [29]. Also, the positively charged aromatic-NPs does not undergo this charge inversion with increasing concentrations of EPS, suggesting that EPS-NPs interactions are not entirely explained by the DLVO theory. As seen in the literature, non-DLVO interactions such as steric forces [30, 31] and surface roughness [32] may play a role in colloidal stability by either increasing or decreasing the energy barrier needed for aggregation. In this case, the insertion of an aromatic group significantly increases the hydrophobicity of the NP surface, as demonstrated by the Rose-Bengal assay (S7 Fig in S1 Appendix), leading to enhanced hydrophobic attractive forces which might have facilitated bridging between particles, thus explaining an aggregation trend even with a reasonably high zeta potential. For carboxylate-NPs, almost no modifications in charge or size were noted for the given concentration range, indicating a high stability even in the presence of various biomolecules in solution.

**Fig 3:**
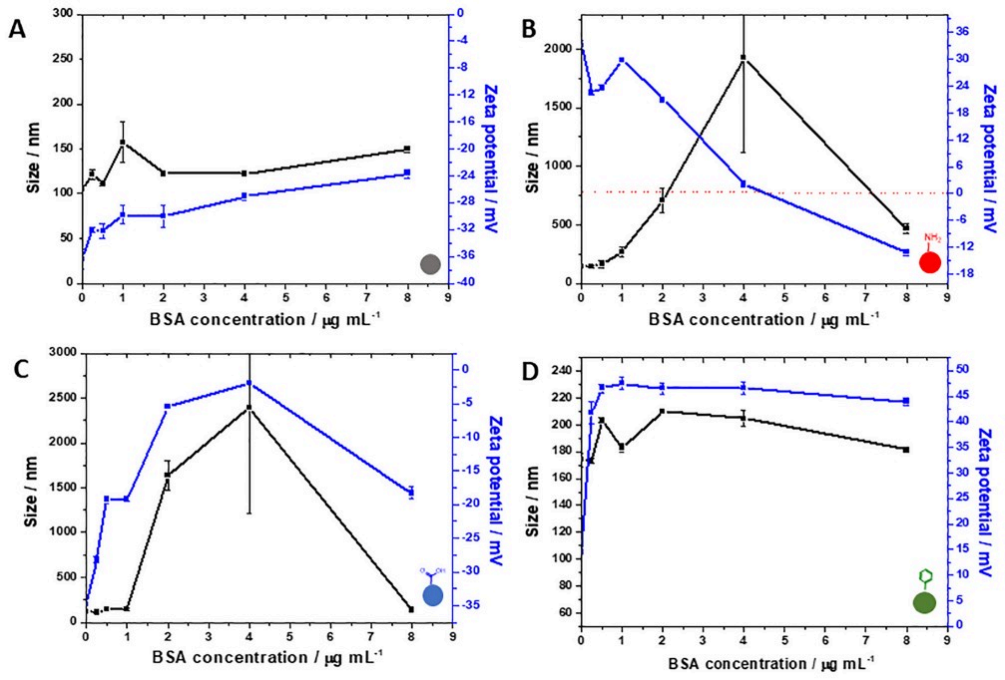
Influence of increasing concentrations of BSA on zeta potential (■) and size (■) of bare (A), amine-functionalised (B), carboxylate-functionalised (C) and aromatic-functionalised (D) silica NPs.

After acquiring these first data, the contributions of proteins and polysaccharides were investigated by using BSA as a protein model and sodium alginate as a polysaccharide model. Solutions of both biomolecules were prepared in the analogous concentration that the respective biochemical group is found in the extracted EPS. Fig 3 depicts the exposure of all NPs to BSA solutions of increasing concentration. The only NP type which does not show the same pattern as seen for EPS exposure is the carboxylate-NPs, in which increasing concentrations of BSA led to a neutralisation of the overall charge. This effect might have been observed due to protein-protein attractive interactions up to a concentration of 4 μg mL^-1^ which induced the formation of aggregates; by doubling this concentration, the excess of proteins in solution possibly led to a prevalence of repulsive interactions thus stabilising the suspension again. This effect is not observed after EPS exposure as the biofilm matrix is rich in various types of biomolecules (not only negatively charged globular proteins such as BSA) which can compete for interactions sites on the NP surface. For amine-NPs, once again a charge inversion is seen by the interaction of the positively charged surface with the overall negatively charged BSA (pI < pH). This observation can suggest a bridging effect that might occur in colloidal suspensions containing proteins. Bridging aggregation happens when proteins with binding sites (in this case, negatively charged sites) on opposite sides act as a bridge between more than one NP [33]. Once again, aromatic-NPs display a slight aggregation trend even with a high positive zeta potential, which confirms the trend seen for the exposure to EPS in which non-DLVO forces could be dictating this effect.

**Fig 4:**
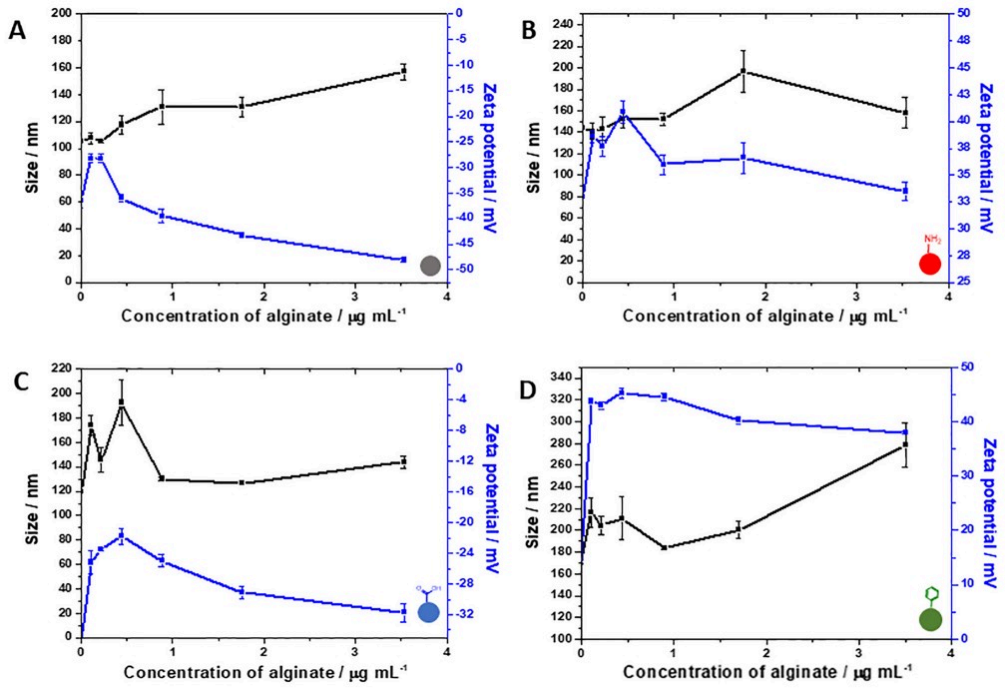
Influence of increasing concentrations of alginate on zeta potential (■) and size (■) of bare (A), amine-functionalised (B), carboxylate-functionalised (C) and aromatic-functionalised (D) silica NPs.

The exposure of the four types of silica NPs to alginate as a model for EPS polysaccharide resulted in very different trends (Fig 4). For bare NPs, alginate forms a consistently larger layer towards higher concentrations, having seen the increase in the size of the NPs. Nevertheless, this increase is not due to aggregation, as the electrostatic stabilisation is also intensified in the presence of the polysaccharide. On the other hand, for amine-NPs, alginate does not seem to interact with the NPs’ surface to the same extent, as the positive charge is only slightly disturbed even in the presence of the negatively charged alginate. This result confirms the previously discussed observations for this NP type that suggest a strong interaction with proteins of the EPS. Carboxylate-NPs shows a pattern of initial aggregation followed by stabilisation and an increase of negative charge with increasing concentrations of alginate. Exposure of carboxylate-NPs to both BSA and alginate does not clearly mimic the exposure to EPS, suggesting that a more complex interaction mechanism takes place. Finally, aromatic-NPs shows a very similar trend to what is seen for exposure to BSA and EPS.

**Fig 5:**
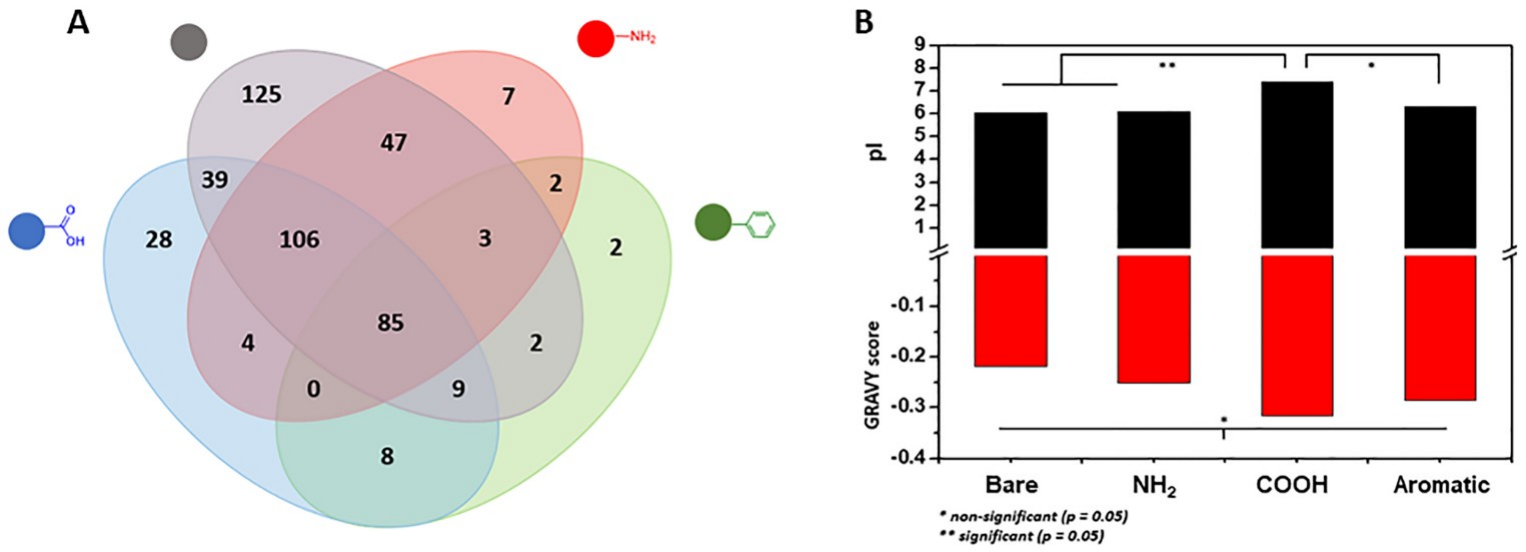
Venn diagram displaying the amount of proteins identified in the protein corona of each type of nanoparticle (A) and average theoretical pI and GRAVY values for the top 20 proteins in terms of LFQ for each nanoparticle type (B). The more positive the GRAVY score, the more hydrophobic nature of the protein.

**Fig 6:**
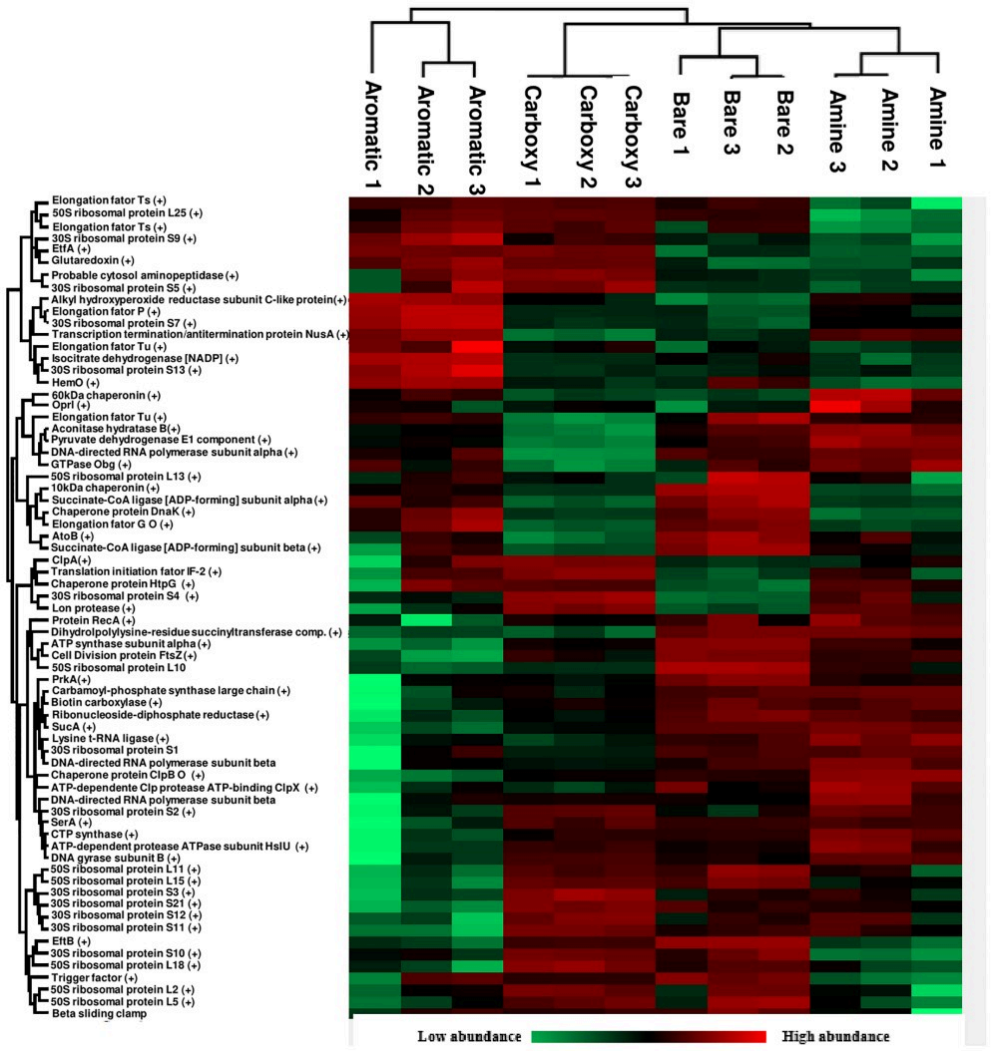
Heat map of label-free quantification (LFQ) of proteins with clustering of nanoparticle samples and proteins, in which red indicates above the average quantification and green indicates below the average quantification for a given sample (symbol “+” shows ANOVA significance; numbers after each nanoparticle type refers to each of the three replicates).

Considering the higher concentration of proteins in the EPS extract in relation to carbohydrates and the protein corona effect that takes place in nanomaterials immersed in any biological media [34–38] a more detailed study was performed in order to elucidate the identity of the proteins that are associated with each NP type. S8 Fig in S1 Appendix shows SDS-PAGE gels of the protein coronas. Interestingly, only the negatively charged NPs displayed a clear set of proteins attached to them whereas almost no proteins are seen in the lanes of the positively charged ones. This observation suggests that the standard sample pre-treatment was not enough to detach the hard protein corona from the surface of these NPs, which is much more likely than the hypothesis that no proteins are associated to these NPs, considering previous shown data and literature on protein corona of positively charged NPs [26, 39, 40] Nevertheless, the proteins attached to bare and carboxylate-NPs seem to be predominantly of high molecular weight. The pattern and intensities of bands in each case are not the same (S8 Fig in S1 Appendix), which is another sign that charge is not the only influencing factor when it comes to interactions with EPS biomolecules. In order to fully comprehend the composition of each protein corona, LC-MS/MS experiments were performed by digesting the protein coronas with trypsin. The data was then treated using the MaxQuant server [41] and statistics were done using the Perseus software platform [42] Fig 5A shows a Venn diagram with all the identified proteins in the corona of each NP type and Fig 5B presents the theoretical pI and GRAVY scores of these proteins. As suggested by the SDS-PAGE results, the bare SNPs contains many more proteins in general (416) and exclusive proteins (125) than any of the other types. Aromatic-NPs had the least number of proteins identified (111), possibly due to the more hydrophobic characteristic given by the aromatic group. Present in all coronas were 85 proteins, which represents 18% of the total number of identified proteins. These numbers, however, relate only to the identified proteins thus disregarding the weight of the contribution of each protein. To obtain a more accurate description of our system, a label-free quantification (LFQ) run in MaxQuant was performed, and the acquired data was treated in the Perseus software. For this step, only proteins present in all the replicates of all samples were considered for a more rigorous statistical analysis. Fig 6 displays a heat map of proteins (horizontal) present in the corona of NPs (columns) in which Euclidean clustering was used to evaluate similarities between replicates. As expected, replicates of the same NP type were clustered together, and Principal Component Analysis (PCA) results also point to a well clustered set of data (S9 Fig in S1 Appendix). Rectangles in red denote higher intensity of a given protein, whereas rectangles in green denote lower intensity. There is a clear variation between the amounts of proteins attached to distinct NP surfaces, which led to a deeper investigation of the most abundant proteins in each corona (S5 Table in S1 Appendix). Some of the proteins are present in the top 20 list of all four types, indicating that for these proteins there might be a lower selectivity in respect to surface chemistry/charge, given that they are present in high abundance regardless of the surface functionalisation. Such proteins include the elongation factor Tu, 60 kDa chaperonin and DNA-directed RNA polymerase subunit beta, for instance. Nevertheless, clear differences were noted and this motivated the verification of the charge and hydrophobicity of each of these proteins, calculated using the ProtParam server for theoretical pI and GRAVY scores, respectively [43].

Interestingly, there were no significant differences amongst the GRAVY values for each NP type even for the aromatic-NPs, which was expected to interact more intensely with more hydrophobic proteins. However, the average pI value of the top 20 proteins found in the corona of carboxylate-NPs is the only one above 7 (7.68), therefore denoting an overall positive charge. This result has statistical significance (p < 0.05) when compared to the coronas of bare (6.41) and amine-functionalised NPs (6.27). The verification that carboxylate-NPs preferentially attract positively charged proteins was further verified with an experiment exposing both bare and carboxylate-NPs to BSA (pI = 4.7) and bovine ribonuclease (pI = 8.93) and running a gel electrophoresis of the protein coronas. The results in S10 Fig in S1 Appendix confirm that carboxylate-NPs interact less intensely with the negatively charged BSA when compared to bare NPs, and that in a mixture of both proteins, the attachment of ribonuclease has a higher preference when compared to bare NPs or a simple protein mixture control. According to Meissner *et al*. [44], for proteins with pI above the pH, the repulsive protein-protein interactions overcome the attractive protein-surface interactions in such way that a monolayer of proteins is usually formed. This hypothesis is confirmed by looking at the stability and non-significant increase in the size of carboxylate-NPs in the presence of EPS (Fig 2), whereas the bare NPs shows an increase of about 40 nm in diameter, which is clearly more than a protein monolayer and in line with the presence of mainly negatively charged proteins. These results also agree with the fact that carboxylate-NPs has a higher uptake into *P*. *fluorescens* biofilms when compared to bare NPs [29]; the interaction with positively charged proteins might have a role in the differential penetration of NPs into the matrix. A qualitative analysis was also done to compare differences in expressions of proteins based on the heat map generated. For instance, the protein cluster in high abundance in amine-NPs and low abundance in aromatic-NPs does not greatly differ in terms of average pI and GRAVY score to the protein cluster with low abundance in amine-NPs and high abundance in aromatic-NPs (pI = 6.17 and GRAVY = -0.224 for the first group and pI = 6.72 and GRAVY = -0.173 for the second). However, when comparing aromatic- to carboxylate-NPs, clear differences are seen; the protein cluster overexpressed in the former has an average pI of 5.84 and average GRAVY score of -0.219, whereas the protein cluster overexpressed in the latter has an average pI of 9.46 and average GRAVY score of -0.419. Therefore, a clear preference of proteins with a more overall positive charge as well as with more hydrophilic nature is existent for carboxylate-NPs.

Differences in protein adsorption profiles for negatively and positively NPs was also observed in a study performed by Paula *et al*. (2014) [26], in which not only surface charge but also surface roughness dictates human blood plasma protein attachment. A study conducted by Puddu and Perry (2012) [45] demonstrated that, at neutral pH, negatively charged bare silica NPs are coated preferentially by cationic polypeptides below a certain concentration threshold and that anionic polypeptides are only adsorbed when the concentration is high enough. Here, this same trend seems to be taking place for carboxylate-NPs but not for bare NPs. Distinct protein corona composition and quantification for two types of surface functionalisation with the same overall charge was also observed for Abdelkhaliq *et al*. (2018) [46]; polystyrene NPs coated with a sulfonated group in general had a diminished protein adsorption capacity when compared to carboxylated polystyrene NPs and less preference for the adsorption of certain classes of proteins.

## Conclusion

### Summary of results

In order to probe interactions between biofilm matrix constitutes and the nanomaterial surfaces, four types of silica NPs (bare, amine-functionalised, carboxylate-functionalised and aromatic-functionalised silica NPs) were synthesised and exposed to EPS extracted from *Pseudomonas fluorescens* biofilms. Dynamic Light Scattering and Zeta potential experiments indicated that NP surface functionalisation influenced the interactions with biomolecules from the EPS. The positively charged NPs were seen to form large aggregates, possibly due to the attachment of negatively charged biomolecules on the NPs surface which either neutralised the overall NP charge or promoting interactions between adjacent NPs leading to aggregation. Regarding the formation of protein corona, the surface chemistry induced a selectivity of the proteins that constitute the corona. Interestingly, carboxylate-NPs seemed to have an increased selectivity towards proteins with a higher isoelectric point.

### Impact and perspectives

The characterisation of biofilms is extremely important for a more tailored approach to NP design. For example, protein-rich biofilms (such as the one used in this study) induce aggregation of positively charged NPs; however, this effect might not be seen in polysaccharide-rich biofilms. The changes in EPS composition may have the potential to have a large influence in the choice of the surface chemistry of nanocarriers and this could be a natural next step for fundamental research on EPS-NP interactions.

The results obtained here, along with previous findings regarding the higher uptake and penetration of carboxylate-NPs, show that the carboxyl-functional group has a significant potential for increasing and tailoring NP-biofilm interactions for specific applications (e.g. nanocarriers of antimicrobials). The findings in relation to NP-biofilm interactions and the formation of the protein corona are of significance and can be used for the rational design of more effective NPs to target biofilms.

## Supporting information

S1 Appendix (DOCX)
