# Supplementary material for: Interactions between functionalised silica nanoparticles and Pseudomonas fluorescens biofilm matrix: A focus on the protein corona
Source: PLoS One. 2020 Jul 23;15(7):e0236441. doi: 10.1371/journal.pone.0236441 (PMC7377396; doi:10.1371/journal.pone.0236441)
Supplement: S1 Appendix — (DOCX) [file pone.0236441.s001.docx]

Interactions between functionalised silica nanoparticles and *Pseudomonas fluorescens* biofilm matrix: a focus on the protein corona

Caio H. N. Barros,^a^ Stephanie Fulaz, ^a^ Stefania Vitale, ^a^ Eoin Casey ^a^ and Laura Quinn ^*a^

^a^School of Chemical and Bioprocess Engineering, University College Dublin, Ireland.

*Corresponding author: [laura.quinn@ucdconnect.ie](mailto:laura.quinn@ucdconnect.ie)

**Table of Contents**

**Table S1.** Size and zeta potential values of functionalised silica nanoparticles……………………………...…3

**Table S2.** Infrared absorption bands assignment for functionalised SNPs……………………………….…..…..3

**Table S3.** Quantification of amine groups using the ninhydrin assay………………………………………………..4

**Table S4.** Characterisation of EPS extract using a cation exchange resin………………………………..………..4

**Table S5.** Image analysis in terms of pixel intensity of protein bands found in SDS-PAGE 10 % performed using ImageJ software……………………………………………………………………………………………………4

**Table S6.** Top 20 proteins in terms of Label-free quantification (LFQ) found in the protein corona of each type of silica nanoparticle……………………………………………………………………………………………………….4

**Table S7.** Full list of proteins identified in the coronas of the four types of silica nanoparticles………6

**Figure S1.** Profile of size distribution by intensity using DLS for bare NPs (A), amino-NPs (B), carboxylate-NPS (C) and aromatic-NPs (D) ……………………………….…………………………………………….……….31

**Figure S2.** FTIR spectra of the synthesised silica nanoparticles ……………………………..….……………..……..32

**Figure S3.** ^1^H NMR spectrum of N1-(3-Trimethoxysilylpropyl) diethylenetriamine (DETA) in D_2_O…....32

**Figure S4.** ^1^H NMR spectrum of succinic anhydride in CDCl_3_…………………………………………….……….……..33

**Figure S5.** ^1^H NMR spectrum of benzoic acid in CDCl_3_………………………………………………………………..……34

**Figure S6.** Dissolution ^1^H NMR spectra of the functionalised silica nanoparticles ………………………..………………………………………………………………………………………………………………………………35

**Figure S7.** Partitioning quotient (amount of Rose-Bengal dye bound to NPs per amount of free dye in solution) as a function of surface area of increasing concentrations of NPs. The steep slope existent only for aromatic-NPs is a clear evidence of increased hydrophobicity …………………………………………………………………………………………………………………………………….………………….35

**Figure S8.** SDS-PAGE 10 % of protein corona of the four types of silica nanoparticles (A) and SDS-PAGE 8 % of the protein corona of bare SNPs and SNPs-COOH ………………………………………………………………..………………………………………………………………………………………36

**Figure S9.** Principal Component Analysis of protein coronas after Label-free Quantification (LFQ) proteomics …………………………………………………………………………………………………………………….……………...36

**Figure S10.** SDS-PAGE 10 % of BSA and ribonuclease in, respectively, pure form (lanes 1-3), in a mixture (1:1) and as protein coronas of bare SNPs (lanes 4-6) and SNPs-COOH (lanes 7-9). …………………………….……………………………………………………………..37

**Table S1.** Size and zeta potential values of functionalised silica nanoparticles

| **Silica NP type** | **Size (SEM*) / nm** | **Size (DLS) / nm** | **Zeta potential / mV** |
| --- | --- | --- | --- |
| Bare | 102.5 ± 20.4 | 105.5 ± 0.9 | -36.4 ± 1.5 |
| Amine | 99.3 ± 21.4 | 143.6 ± 1.7 | +33.0 ± 1.0 |
| Carboxylate | 98.0 ± 19.9 | 119.9 ± 1.1 | -34.8 ± 0.9 |
| Aromatic | 116.5 ± 21.7 | 174.0 ± 4.8 | +14.6 ± 0.5 |

*At least 3 images and 100 nanoparticles were considered

**Table S2.** Infrared absorption bands assignment for functionalised SNPs

| **Vibrational mode** | **Bare** | **NH_2_** | **COOH** | **Aromatic** |
| --- | --- | --- | --- | --- |
| Si-O-Si sym. stretching | 841 cm^-1^ | 840 cm^-1^ | 838 cm^-1^ | Approx. 830 cm^-1^ |
| Si-OH stretching | 988 cm^-1^ | 988 cm^-1^ | 982 cm^-1^ | 986 cm^-1^ |
| Si-O-Si asym. stretching | Approx. 1120 cm^-1^ | Approx. 1100 cm^-1^ | Approx. 1100 cm^-1^ | 1111 cm^-1^ |
| O-H stretching | 3415 cm^-1^ | 3382 cm^-1^ | 3375 cm^-1^ | 3390 cm^-1^ |
| O-H bending (out of plane) | 672 cm^-1^ | 681 cm^-1^ | 674 cm^-1^ | 673 cm^-1^ |
| O-H bending physiosorbed water | 1546 cm^-1^ | 1551 cm^-1^ | 1564 cm^-1^ | 1564 cm^-1^ |
| C-H stretching |  | 2947 cm^-1^ | 2948 cm^-1^ | 2964 cm^-1^ |
| C=O stretching |  |  | 1564 cm^-1^ | 1616 cm^-1^ |
| C-O stretching |  |  | 1297 cm^-1^ |  |

**Table S3.** Quantification of amine groups using the ninhydrin assay

| **Description** | **NH_2_ groups / nm^2^** |
| --- | --- |
| SNPs | 0 |
| Amine-NPs | 1.62 |
| Carboxylate-NPs | 0.95 |

**Table S4.** Characterisation of EPS extract using a cation exchange resin

| **Average biofilm mass (g)** | **[Proteins] / mg/g of biofilm** | **[Carbohydrates] / mg/g of biofilm** |
| --- | --- | --- |
| 0.831 ± 0.006 | 0.377 ± 0.071 | 0.129 ± 0.063 |

**Table S5.** Image analysis in terms of pixel intensity of protein bands found in SDS-PAGE 10 % performed using ImageJ software

|  | **BSA** | **Rib** | **BSA+ Rib** | **SNP + BSA** | **SNP + Rib** | **SNP + BSA + Rib** | **SNP-COOH + BSA** | **SNP-COOH + rib** | **SNP-COOH + BSA + Rib** |
| --- | --- | --- | --- | --- | --- | --- | --- | --- | --- |
| **Int. BSA (a.u.)** | 95535.48 | 0 | 107137.5 | 107137.5 | 0 | 81092.82 | 72525.36 | 0 | 71346.01 |
| **Int. Rib (a.u.)** | 0 | 10763.31 | 12657.5 | 0 | 28600.61 | 29084.98 | 0 | 14745.1 | 42077.84 |
| **% BSA in mix** |  |  | 89.4 |  |  | 73.6 |  |  | 62.9 |
| **% Rib in mix** |  |  | 10.6 |  |  | 26.4 |  |  | 37.1 |
| **% increase in mix (BSA)** |  |  | 12.1 |  |  | -24.3 |  |  | -1.6 |
| **% increase in mix (Rib)** |  |  | 17.6 |  |  | 1.7 |  |  | 185.4 |

**Table S6.** Top 20 proteins in terms of Label-free quantification (LFQ) found in the protein corona of each type of silica nanoparticle

|  | **Bare** | **NH_2_** | **COOH** | **Aromatic** |
| --- | --- | --- | --- | --- |
| **1** | 10 kDa chaperonin | Elongation factor Tu | Elongation factor Tu | Elongation factor Tu |
| **2** | Elongation factor Tu | Chaperone protein ClpB O | DNA-directed RNA polymerase subunit beta | Chaperone protein DnaK |
| **3** | Chaperone protein DnaK | DNA-directed RNA polymerase subunit beta | 30S ribosomal protein S4 | DNA-directed RNA polymerase subunit beta |
| **4** | Chaperone protein ClpB O | DNA-directed RNA polymerase subunit beta | Chaperone protein ClpB O | 60 kDa chaperonin |
| **5** | DNA-directed RNA polymerase subunit beta | 60 kDa chaperonin | 30S ribosomal protein S2 | 30S ribosomal protein S4 |
| **6** | DNA-directed RNA polymerase subunit beta | SucA | DNA-directed RNA polymerase subunit beta | DNA-directed RNA polymerase subunit beta |
| **7** | Dihydrolipoyllysine-residue succinyltransferase component of 2-oxoglutarate dehydrogenase complex | 30S ribosomal protein S4 | 30S ribosomal protein S3 | Chaperone protein ClpB O |
| **8** | SucA | 30S ribosomal protein S2 | Acetyltransferase component of pyruvate dehydrogenase complex | Acyl carrier protein |
| **9** | 30S ribosomal protein S1 | Dihydrolipoyllysine-residue succinyltransferase component of 2-oxoglutarate dehydrogenase complex | 60 kDa chaperonin | 30S ribosomal protein S7 |
| **10** | EtfB | 30S ribosomal protein S1 | Chaperone protein DnaK | 30S ribosomal protein S1 |
| **11** | 30S ribosomal protein S4 | Carbamoyl-phosphate synthase large chain | DNA-binding protein HU-beta | 30S ribosomal protein S13 |
| **12** | 60 kDa chaperonin | PvdL | 30S ribosomal protein S21 | Elongation factor G O |
| **13** | ATP synthase subunit alpha | 30S ribosomal protein S3 | 30S ribosomal protein S10 | Elongation factor Ts |
| **14** | 30S ribosomal protein S2 | Chaperone protein DnaK | 30S ribosomal protein S11 | Transcription termination/antitermination protein NusA |
| **15** | 30S ribosomal protein S10 | Acetyltransferase component of pyruvate dehydrogenase complex | EtfB | 30S ribosomal protein S2 |
| **16** | Trigger factor | DNA-directed RNA polymerase subunit alpha | 30S ribosomal protein S1 | 30S ribosomal protein S9 |
| **17** | 30S ribosomal protein S3 | ATP synthase subunit alpha | Translation initiation factor IF-3 | DNA-directed RNA polymerase subunit alpha |
| **18** | Carbamoyl-phosphate synthase large chain | 30S ribosomal protein S11 | 30S ribosomal protein S12 | Trigger factor |
| **19** | Acetyltransferase component of pyruvate dehydrogenase complex O | Pyruvate dehydrogenase E1 component | Elongation factor Ts | 30S ribosomal protein S3 |
| **20** | PrkA | ATP-dependent protease ATPase subunit HslU | Translation initiation factor IF-2 | 30S ribosomal protein S10 |

**Table S7.** Full list of proteins identified in the coronas of the four types of silica nanoparticles

| **Bare NPs** | |
| --- | --- |
| **UNIPROT Code** | **Protein name** |
| G8PW44 | Beta sliding clamp |
| G8PW46 | DNA gyrase subunit B |
| G8PW52 | Glycine--tRNA ligase beta subunit |
| G8PW57 | Proline--tRNA ligase |
| G8PW62 | dCTP deaminase |
| G8PW68 | Lysine-arginine-ornithine-binding periplasmic protein |
| G8PWB1 | Arginine deiminase |
| G8PWB6 | HemO |
| G8PWC1 | Phosphogluconate dehydratase |
| G8PWD3 | Glucose-6-phosphate 1-dehydrogenase |
| G8PWD4 | 6-phosphogluconolactonase |
| G8PWF1 | Diaminobutyrate--2-oxoglutarate aminotransferase |
| G8PWR0 | Xaa-His dipeptidase |
| G8PWV7 | 30S ribosomal protein S20 |
| G8PWW0 | Glutamate 5-kinase |
| G8PWW1 | GTPase Obg |
| G8PWW3 | 50S ribosomal protein L21 |
| G8PWX0 | Endopeptidase La |
| G8PWX6 | ABC transporter, ATP-binding protein |
| G8PX52 | DNA polymerase I |
| G8PX62 | AlgB |
| G8PX85 | Qor |
| G8PX90 | Methionyl-tRNA formyltransferase |
| G8PXB9 | Long-chain-fatty-acid--CoA ligase |
| G8PXD9 | Iron-regulated protein A |
| G8PXE7 | RNA polymerase-associated protein RapA |
| G8PXG7 | MexC |
| G8PXI9 | Alcohol dehydrogenase II |
| G8PXN5 | Inosine-5-monophosphate dehydrogenase |
| G8PXS7 | Methylglutaconyl-CoA hydratase |
| G8PXS8 | Methylcrotonyl-CoA carboxylase biotin-containing subunit |
| G8PXU5 | Probable transcriptional regulatory protein PSF113_3903 |
| G8PXX0 | AMP nucleosidase |
| G8PXX1 | Acyl-CoA dehydrogenase |
| G8PXX5 | Glutamate-1-semialdehyde 2,1-aminomutase |
| G8PXX9 | Phosphate starvation-inducible ATPase PhoH with RNA binding motif |
| G8PXY1 | CorC |
| G8PXZ1 | Lipoyl synthase |
| G8PY00 | Ribosomal silencing factor RsfS |
| G8PY19 | Hpa2 |
| G8PY77 | Luciferase-like protein |
| G8PY94 | GcdH |
| G8PYD7 | TctC |
| G8PYE9 | Periplasmic serine endoprotease DegP-like |
| G8PYF8 | Phosphoribosylaminoimidazole-succinocarboxamide synthase |
| G8PYK4 | Thiol peroxidase |
| G8PYR7 | DNA-binding protein HU-beta |
| G8PYR8 | Lon protease |
| G8PYR9 | ATP-dependent Clp protease ATP-binding subunit ClpX |
| G8PYS1 | Trigger factor |
| G8PYT2 | Uncharacterized protein |
| G8PYX1 | Soluble pyridine nucleotide transhydrogenase |
| G8PYX4 | Glyceraldehyde-3-phosphate dehydrogenase |
| G8PYX5 | Transcription-repair-coupling factor |
| G8PYY0 | FadQ |
| G8PYY4 | Uncharacterized protein |
| G8PYY5 | DNA topoisomerase 1 |
| G8PYY8 | Fatty acid oxidation complex subunit alpha |
| G8PZ16 | Uncharacterized protein |
| G8PZ31 | GTP cyclohydrolase-2 |
| G8PZ35 | Transcription antitermination protein NusB |
| G8PZ64 | Single-stranded DNA-binding protein |
| G8PZ66 | UvrABC system protein A |
| G8PZ69 | 50S ribosomal protein L17 |
| G8PZ70 | DNA-directed RNA polymerase subunit alpha |
| G8PZ71 | 30S ribosomal protein S4 |
| G8PZ72 | 30S ribosomal protein S11 |
| G8PZ73 | 30S ribosomal protein S13 |
| G8PZ76 | GabD |
| G8PZE7 | PtsP |
| G8PZF9 | ATPase associated with various cellular activities, AAA_5 |
| G8PZI4 | SrmB |
| G8PZL2 | SlyA |
| G8PZM4 | Aspartate aminotransferase/Aromatic-amino-acid aminotransferase |
| G8PZM7 | PhhR |
| G8PZQ4 | KaiC |
| G8PZW2 | Glyceraldehyde-3-phosphate dehydrogenase, putative |
| G8PZY7 | Coproporphyrinogen-III oxidase |
| G8PZZ7 | DNA ligase |
| G8Q000 | Transcriptional regulator, GntR family |
| G8Q005 | Transcriptional regulator, GntR family |
| G8Q010 | Ribosomal RNA large subunit methyltransferase K/L |
| G8Q020 | Cation abc transporter periplasmic cation-binding protein |
| G8Q025 | 2-methylcitrate dehydratase FeS dependent |
| G8Q038 | Oxidoreductase, short chain dehydrogenase/reductase family |
| G8Q048 | 50S ribosomal protein L15 |
| G8Q049 | 50S ribosomal protein L30 |
| G8Q050 | 30S ribosomal protein S5 |
| G8Q051 | 50S ribosomal protein L18 |
| G8Q052 | RplF |
| G8Q053 | RpsH |
| G8Q054 | 30S ribosomal protein S14 |
| G8Q055 | 50S ribosomal protein L5 |
| G8Q056 | 50S ribosomal protein L24 |
| G8Q057 | 50S ribosomal protein L14 |
| G8Q058 | 30S ribosomal protein S17 |
| G8Q059 | 50S ribosomal protein L29 |
| G8Q060 | 50S ribosomal protein L16 |
| G8Q061 | 30S ribosomal protein S3 |
| G8Q064 | 50S ribosomal protein L2 |
| G8Q065 | 50S ribosomal protein L23 |
| G8Q066 | 50S ribosomal protein L4 |
| G8Q067 | 50S ribosomal protein L3 |
| G8Q068 | 30S ribosomal protein S10 |
| G8Q069 | Elongation factor Tu |
| G8Q070 | Elongation factor G |
| G8Q071 | 30S ribosomal protein S7 |
| G8Q072 | 30S ribosomal protein S12 |
| G8Q073 | DNA-directed RNA polymerase subunit beta |
| G8Q074 | DNA-directed RNA polymerase subunit beta |
| G8Q075 | 50S ribosomal protein L7/L12 |
| G8Q076 | 50S ribosomal protein L10 |
| G8Q077 | 50S ribosomal protein L1 |
| G8Q078 | 50S ribosomal protein L11 |
| G8Q079 | Transcription termination/antitermination protein NusG |
| G8Q081 | Elongation factor Tu |
| G8Q085 | Tyrosine--tRNA ligase |
| G8Q091 | Dioxygenases related to 2-nitropropane dioxygenase |
| G8Q0C0 | SerA |
| G8Q0P8 | Protein-glutamate methylesterase/protein-glutamine glutaminase |
| G8Q0S5 | Asparagine synthetase |
| G8Q0U3 | Penicillin amidase family protein |
| G8Q0U6 | Delta 1-piperideine-2-carboxylate reductase / Delta 1-pyrroline-2-carboxylate reductase |
| G8Q0Z6 | Acyl-CoA synthetase(AMP-forming)/AMP-acid ligase |
| G8Q101 | Butyryl-CoA dehydrogenase |
| G8Q151 | EtfA |
| G8Q152 | EtfB |
| G8Q186 | 50S ribosomal protein L32 |
| G8Q188 | Chaperone SurA |
| G8Q194 | PrkA |
| G8Q197 | Multifunctional CCA protein |
| G8Q1A2 | 30S ribosomal protein S21 |
| G8Q1A4 | RNA polymerase sigma factor RpoD |
| G8Q1B3 | Lysine 2-monooxygenase |
| G8Q1C3 | Acyl-CoA dehydrogenase |
| G8Q1C6 | Acyl-CoA dehydrogenase |
| G8Q1I6 | Carboxyl-terminal protease |
| G8Q1K1 | TypA/BipA |
| G8Q1K6 | Fructose-1,6-bisphosphatase class 1 |
| G8Q1M7 | Glucans biosynthesis protein G |
| G8Q1P0 | Ubiquinone/menaquinone biosynthesis C-methyltransferase UbiE |
| G8Q1P3 | PhaF |
| G8Q1P9 | Uncharacterized protein |
| G8Q1R2 | DNA gyrase subunit A |
| G8Q1R4 | PheA |
| G8Q1R8 | 30S ribosomal protein S1 |
| G8Q1W2 | OprI |
| G8Q1W4 | Phospho-2-dehydro-3-deoxyheptonate aldolase |
| G8Q1W8 | CysB |
| G8Q1X2 | Elongation factor P |
| G8Q262 | Ribonuclease E |
| G8Q2D2 | Chaperone protein HtpG |
| G8Q2G8 | NAD/NADP-dependent betaine aldehyde dehydrogenase |
| G8Q2I4 | Fructose-1,6-bisphosphate aldolase |
| G8Q2I7 | Phosphoglycerate kinase |
| G8Q2J1 | S-adenosylmethionine synthase |
| G8Q2K3 | Adenosylhomocysteinase |
| G8Q2K4 | Methylenetetrahydrofolate reductase |
| G8Q2K7 | ATP-dependent RNA helicase RhlE |
| G8Q2K8 | UPF0312 protein PSF113_5506 |
| G8Q2L8 | ATP-dependent protease ATPase subunit HslU |
| G8Q2M3 | 50S ribosomal protein L31 |
| G8Q2N5 | GltB |
| G8Q2N6 | GltE |
| G8Q2V1 | ATP-dependent RNA helicase DeaD |
| G8Q2V5 | Aspartate/tyrosine/aromatic aminotransferase |
| G8Q2W0 | Putative ATP/GTP-binding protein |
| G8Q2Z3 | Siderophore biosynthesis protein |
| G8Q2Z5 | PvdL |
| G8Q302 | PvdH |
| G8Q307 | Phosphoenolpyruvate synthase |
| G8Q314 | OprF |
| G8Q3A8 | Putative lipoprotein |
| G8Q3B1 | Succinate--CoA ligase [ADP-forming] subunit alpha |
| G8Q3B2 | Succinate--CoA ligase [ADP-forming] subunit beta |
| G8Q3B3 | Dihydrolipoyl dehydrogenase |
| G8Q3B4 | Dihydrolipoyllysine-residue succinyltransferase component of 2-oxoglutarate dehydrogenase complex |
| G8Q3B5 | SucA |
| G8Q3B6 | SdhB |
| G8Q3B7 | Succinate dehydrogenase flavoprotein subunit |
| G8Q3E7 | Chorismate synthase |
| G8Q3K6 | Glutathione synthetase |
| G8Q3L2 | PyrC |
| G8Q3N9 | Thiosulfate sulfurtransferase, rhodanese |
| G8Q3P4 | Phosphopantetheine adenylyltransferase |
| G8Q3Q0 | Dihydroxy-acid dehydratase |
| G8Q3S2 | Bifunctional protein PutA |
| G8Q3S7 | Ribosomal RNA large subunit methyltransferase J |
| G8Q3S8 | Peptide methionine sulfoxide reductase MsrA |
| G8Q3T1 | Acetyltransferase component of pyruvate dehydrogenase complex |
| G8Q3T2 | Pyruvate dehydrogenase E1 component |
| G8Q3V3 | Bifunctional protein HldE |
| G8Q3V6 | Oxidoreductase, FAD-binding protein |
| G8Q3X1 | DNA topoisomerase 4 subunit B |
| G8Q3X4 | DNA topoisomerase 4 subunit A |
| G8Q3X7 | Phosphoserine phosphatase |
| G8Q3Y0 | Sulfurtransferase |
| G8Q3Z3 | Hfq |
| G8Q3Z5 | Protein HflK |
| G8Q448 | Transcription factor jumonji |
| G8Q451 | PvdD |
| G8Q452 | PvdJ1 |
| G8Q453 | PvdJ2 |
| G8Q454 | Non-ribosomal peptide synthetase modules, amino acid adenylation, pyoverdine |
| G8Q456 | Pyoverdine sidechain peptide synthetase l-thr-l-ser component |
| G8Q459 | PvdN |
| G8Q4F2 | Universal stress protein family 5 |
| G8Q4I4 | Site-determining protein |
| G8Q4K0 | Tail-specific protease |
| G8Q4K7 | Peptidyl-prolyl cis-trans isomerase |
| G8Q4L0 | Recombination-associated protein RdgC |
| G8Q4R2 | SpuD/PotF1 |
| G8Q4R4 | SpuB |
| G8Q4R6 | SpuI |
| G8Q4T4 | Amino-acid acetyltransferase |
| G8Q4T5 | Acetylornithine deacetylase |
| G8Q4T6 | Adenylate cyclase |
| G8Q4U4 | Aminomethyltransferase |
| G8Q4W1 | Ribonuclease R |
| G8Q4W2 | 23S rRNA (guanosine-2-O-)-methyltransferase RlmB |
| G8Q4W3 | 30S ribosomal protein S6 |
| G8Q4W4 | 30S ribosomal protein S18 |
| G8Q4W6 | 50S ribosomal protein L9 |
| G8Q4X7 | NH(3)-dependent NAD(+) synthetase |
| G8Q4Y0 | L-pipecolate dehydrogenase |
| G8Q4Y7 | Short-chain dehydrogenase reductase sdr |
| G8Q503 | CbpA |
| G8Q539 | PvdA |
| G8Q568 | Aminotransferase |
| G8Q570 | Glutamate--tRNA ligase |
| G8Q575 | 16 kDa heat shock protein A |
| G8Q591 | Amidophosphoribosyltransferase |
| G8Q5L6 | Phosphoribosylformylglycinamidine cyclo-ligase |
| G8Q5M3 | RelA |
| G8Q5P8 | Ribonucleoside-diphosphate reductase |
| G8Q5R9 | AmrZ |
| G8Q5S8 | Transcription termination factor Rho |
| G8Q5T5 | Ppx |
| G8Q5T6 | Polyphosphate kinase |
| G8Q5V8 | AlgR |
| G8Q5Z9 | D-amino acid dehydrogenase |
| G8Q609 | Biotin carboxylase |
| G8Q610 | Biotin carboxyl carrier protein of acetyl-CoA carboxylase |
| G8Q617 | Biosynthetic arginine decarboxylase |
| G8Q6C4 | Exodeoxyribonuclease III |
| G8Q6D4 | Glutamine--tRNA ligase |
| G8Q6F4 | Threonine--tRNA ligase |
| G8Q6F5 | Translation initiation factor IF-3 |
| G8Q6F7 | 50S ribosomal protein L20 |
| G8Q6F8 | Phenylalanine--tRNA ligase alpha subunit |
| G8Q6H7 | AtoB |
| G8Q6I2 | General stress protein |
| G8Q6L2 | Sulfite reductase [NADPH] hemoprotein beta-component |
| G8Q6P2 | Aspartokinase |
| G8Q6P3 | Alanine--tRNA ligase |
| G8Q6R1 | Ribonucleoside-diphosphate reductase subunit beta |
| G8Q6W0 | 60 kDa chaperonin |
| G8Q6W1 | 10 kDa chaperonin |
| G8Q6W6 | Methionine--tRNA ligase |
| G8Q6X2 | Aldehyde dehydrogenase |
| G8Q6X4 | 50S ribosomal protein L33 |
| G8Q6X5 | RpmB |
| G8Q6X6 | Dipeptide-binding ABC transporter, periplasmic substrate-binding component |
| G8Q6X8 | Coenzyme A biosynthesis bifunctional protein CoaBC |
| G8Q6Y0 | AlgC |
| G8Q6Y1 | Acetylglutamate kinase |
| G8Q6Z8 | OxyR |
| G8Q717 | ImpC |
| G8Q718 | ImpD |
| G8Q724 | ClpB |
| G8Q7C8 | Phenylalanine--tRNA ligase beta subunit |
| G8Q7V1 | Alkyl hydroperoxide reductase subunit C-like protein |
| G8Q7W0 | Cys-tRNA(Pro)/Cys-tRNA(Cys) deacylase |
| G8Q7W4 | Glycerol-3-phosphate dehydrogenase |
| G8Q7X8 | SdaA |
| G8Q7X9 | Aminomethyltransferase (Glycine cleavage system T protein) |
| G8Q7Z1 | Cell division coordinator CpoB |
| G8Q7Z3 | Tol-Pal system protein TolB |
| G8Q7Z7 | 4-hydroxybenzoyl-thioesterase |
| G8Q802 | Aspartate--tRNA(Asp/Asn) ligase |
| G8Q812 | UPF0234 protein PSF113_4629 |
| G8Q838 | OadA |
| G8Q839 | AccC |
| G8Q844 | DNA helicase |
| G8Q893 | Glutamine--fructose-6-phosphate aminotransferase [isomerizing] |
| G8Q895 | Bifunctional protein GlmU |
| G8Q897 | ATP synthase subunit beta |
| G8Q8E0 | Chaperone protein DnaK |
| G8Q8E1 | Chaperone protein DnaJ |
| G8Q8E2 | 4-hydroxy-tetrahydrodipicolinate reductase |
| G8Q8E3 | Carbamoyl-phosphate synthase small chain |
| G8Q8E4 | Carbamoyl-phosphate synthase large chain |
| G8Q8E7 | Rna-binding protein containing kh domain |
| G8Q8E9 | ATP-dependent zinc metalloprotease FtsH |
| G8Q8F1 | Phosphoglucosamine mutase |
| G8Q8F4 | Transcription termination/antitermination protein NusA |
| G8Q8F5 | Translation initiation factor IF-2 |
| G8Q8F6 | Ribosome-binding factor A |
| G8Q8H4 | SpeC |
| G8Q8M6 | NADH-quinone oxidoreductase |
| G8Q8M7 | NADH-quinone oxidoreductase subunit F |
| G8Q8M9 | NADH-quinone oxidoreductase subunit C/D |
| G8Q8N7 | Adenylosuccinate lyase |
| G8Q8P1 | Isocitrate dehydrogenase [NADP] |
| G8Q8P2 | Isocitrate dehydrogenase [NADP] |
| G8Q8P5 | ClpA |
| G8Q8Q4 | Serine--tRNA ligase |
| G8Q8Y7 | Protein translocase subunit SecA |
| G8Q8Y9 | Uncharacterized protein |
| G8Q8Z4 | Formyltetrahydrofolate deformylase |
| G8Q8Z5 | MvaT |
| G8Q906 | Pyruvate kinase |
| G8Q931 | Two-component response regulator |
| G8Q953 | Gcd |
| G8Q963 | Phosphoribosylformylglycinamidine synthase |
| G8Q967 | ATP synthase gamma chain |
| G8Q968 | ATP synthase subunit alpha |
| G8Q970 | ATP synthase subunit b |
| G8Q974 | ParB |
| G8Q977 | tRNA uridine 5-carboxymethylaminomethyl modification enzyme MnmG |
| G8Q985 | Peptide chain release factor 3 |
| G8Q9B1 | Glutamyl-tRNA(Gln) amidotransferase subunit A |
| G8Q9B3 | MreB |
| G8Q9C0 | TldD |
| G8Q9C1 | UPF0307 protein PSF113_0931 |
| G8Q9D4 | Lipopolysaccharide export system protein LptA |
| G8Q9E4 | UDP-N-acetylglucosamine 1-carboxyvinyltransferase |
| G8Q9F4 | Medium-chain-fatty-acid-CoA ligase |
| G8Q9F6 | Putative exported protein |
| G8Q9X6 | Elongation factor 4 |
| G8Q9X9 | Probable cytosol aminopeptidase |
| G8Q9Y2 | Valine--tRNA ligase |
| G8Q9Y4 | DNA-binding protein HU, form N |
| G8QA05 | Cyclic pyranopterin monophosphate synthase |
| G8QA09 | GDP-mannose 6-dehydrogenase |
| G8QA19 | AlgF |
| G8QA20 | Mannose-6-phosphate isomerase / Mannose-1-phosphate guanylyltransferase (GDP) |
| G8QA35 | Cell division protein FtsZ |
| G8QA36 | Cell division protein FtsA |
| G8QA38 | D-alanine--D-alanine ligase |
| G8QA39 | UDP-N-acetylmuramate--L-alanine ligase |
| G8QA42 | UDP-N-acetylmuramoylalanine--D-glutamate ligase |
| G8QA44 | UDP-N-acetylmuramoyl-tripeptide--D-alanyl-D-alanine ligase |
| G8QA45 | UDP-N-acetylmuramoyl-L-alanyl-D-glutamate--2,6-diaminopimelate ligase |
| G8QA48 | Ribosomal RNA small subunit methyltransferase H |
| G8QA89 | Inositol-1-monophosphatase |
| G8QA93 | Cysteine desulfurase IscS |
| G8QA97 | Chaperone protein HscA homolog |
| G8QAA0 | Nucleoside diphosphate kinase |
| G8QAA4 | 4-hydroxy-3-methylbut-2-en-1-yl diphosphate synthase (flavodoxin) |
| G8QAA5 | Histidine--tRNA ligase |
| G8QAA8 | GTPase Der |
| G8QAB4 | Exodeoxyribonuclease 7 large subunit |
| G8QAB7 | Inosine-5-monophosphate dehydrogenase |
| G8QAG6 | Decarboxylase family protein |
| G8QAI3 | UTP--glucose-1-phosphate uridylyltransferase |
| G8QAI9 | MvaU |
| G8QAJ5 | Oxidoreductase, aldo/keto reductase family |
| G8QAL3 | Branched-chain-amino-acid aminotransferase |
| G8QAM8 | 3-oxoacyl-[acyl-carrier protein] reductase |
| G8QAV2 | 30S ribosomal protein S9 |
| G8QAV3 | 50S ribosomal protein L13 |
| G8QAV8 | Tryptophan--tRNA ligase |
| G8QB10 | Fumarylacetoacetase |
| G8QB27 | Lon protease |
| G8QB32 | 30S ribosomal protein S16 |
| G8QB35 | 50S ribosomal protein L19 |
| G8QB38 | Homoserine dehydrogenase |
| G8QB39 | ThrC |
| G8QB66 | Lysine--tRNA ligase |
| G8QB72 | Outer membrane lipoprotein omp16 |
| G8QB87 | Putative exported protein |
| G8QB98 | 2,3,4,5-tetrahydropyridine-2,6-dicarboxylate N-succinyltransferase |
| G8QB99 | Uncharacterized protein |
| G8QBA6 | Methionine aminopeptidase |
| G8QBA8 | Dihydrolipoamide acetyltransferase component of pyruvate dehydrogenase complex |
| G8QBM9 | Nadph-dependent fmn reductase |
| G8QBN2 | NAD kinase |
| G8QBP7 | PggL |
| G8QBT1 | Phosphate acetyltransferase |
| G8QBT3 | Multifunctional fusion protein |
| G8QBT4 | Sulfate adenylyltransferase subunit 2 |
| G8QBT8 | NikK |
| G8QBU5 | Putative lipoprotein |
| G8QBU7 | Probable malate:quinone oxidoreductase |
| G8QBU9 | Uncharacterized protein |
| G8QBX2 | Ribose-phosphate pyrophosphokinase |
| G8QBX3 | 50S ribosomal protein L25 |
| G8QBX4 | Peptidyl-tRNA hydrolase |
| G8QBZ6 | Uncharacterized protein |
| G8QBZ7 | 30S ribosomal protein S2 |
| G8QBZ8 | Elongation factor Ts |
| G8QC00 | Ribosome-recycling factor |
| G8QC01 | Ditrans,polycis-undecaprenyl-diphosphate synthase ((2E,6E)-farnesyl-diphosphate specific) |
| G8QC06 | Outer membrane chaperone skp |
| G8QC07 | UDP-3-O-acylglucosamine N-acyltransferase |
| G8QC12 | DNA-directed DNA polymerase |
| G8QC13 | Acetyl-coenzyme A carboxylase carboxyl transferase subunit alpha |
| G8QC15 | CTP synthase |
| G8QC16 | 2-dehydro-3-deoxyphosphooctonate aldolase |
| G8QC17 | Enolase |
| G8QC21 | S-(Hydroxymethyl)glutathione dehydrogenase |
| G8QC31 | DNA mismatch repair protein MutS |
| G8QC56 | Protein RecA |
| G8QC58 | Decarboxylase family protein |
| G8QC65 | Ferredoxin--NADP(+) reductase |
| G8QC75 | Aconitate hydratase B |
| G8QCG4 | GdhB |
| G8QCN6 | Chromosome segregation ATPase |
| G8QCP5 | Acetolactate synthase |
| G8QCQ0 | Uncharacterized protein |
| G8QCQ6 | Periplasmic hemin-binding protein |
| G8QCR4 | CbrB |
| G8QCR8 | Pantothenate synthetase |
| G8QCU1 | Chaperone protein ClpB |
| G8QCU3 | Pseudouridine synthase |
| **Amine-NPs** | |
| **UNIPROT Code** | **Protein name** |
| G8PW44 | Beta sliding clamp |
| G8PW46 | DNA gyrase subunit B |
| G8PW52 | Glycine--tRNA ligase beta subunit |
| G8PW57 | Proline--tRNA ligase |
| G8PWB1 | Arginine deiminase |
| G8PWB6 | HemO |
| G8PWW0 | Glutamate 5-kinase |
| G8PWW1 | GTPase Obg |
| G8PWX0 | Endopeptidase La |
| G8PWX6 | ABC transporter, ATP-binding protein |
| G8PWX8 | Serine hydroxymethyltransferase |
| G8PXD9 | Iron-regulated protein A |
| G8PXE7 | RNA polymerase-associated protein RapA |
| G8PXI9 | Alcohol dehydrogenase II |
| G8PXS7 | Methylglutaconyl-CoA hydratase |
| G8PXX5 | Glutamate-1-semialdehyde 2,1-aminomutase |
| G8PXX9 | Phosphate starvation-inducible ATPase PhoH with RNA binding motif |
| G8PXY1 | CorC |
| G8PY94 | GcdH |
| G8PYE5 | L-aspartate oxidase |
| G8PYE9 | Periplasmic serine endoprotease DegP-like |
| G8PYF8 | Phosphoribosylaminoimidazole-succinocarboxamide synthase |
| G8PYR7 | DNA-binding protein HU-beta |
| G8PYR8 | Lon protease |
| G8PYR9 | ATP-dependent Clp protease ATP-binding subunit ClpX |
| G8PYS1 | Trigger factor |
| G8PYX0 | AcdB |
| G8PYX4 | Glyceraldehyde-3-phosphate dehydrogenase |
| G8PYX5 | Transcription-repair-coupling factor |
| G8PZ37 | 3,4-dihydroxy-2-butanone 4-phosphate synthase |
| G8PZ64 | Single-stranded DNA-binding protein |
| G8PZ66 | UvrABC system protein A |
| G8PZ69 | 50S ribosomal protein L17 |
| G8PZ70 | DNA-directed RNA polymerase subunit alpha |
| G8PZ71 | 30S ribosomal protein S4 |
| G8PZ72 | 30S ribosomal protein S11 |
| G8PZ73 | 30S ribosomal protein S13 |
| G8PZE7 | PtsP |
| G8PZI4 | SrmB |
| G8PZL0 | Yeca family protein |
| G8PZM4 | Aspartate aminotransferase/Aromatic-amino-acid aminotransferase |
| G8PZQ4 | KaiC |
| G8PZW2 | Glyceraldehyde-3-phosphate dehydrogenase, putative |
| G8PZY7 | Coproporphyrinogen-III oxidase |
| G8PZZ7 | DNA ligase |
| G8Q005 | Transcriptional regulator, GntR family |
| G8Q010 | Ribosomal RNA large subunit methyltransferase K/L |
| G8Q025 | 2-methylcitrate dehydratase FeS dependent |
| G8Q048 | 50S ribosomal protein L15 |
| G8Q050 | 30S ribosomal protein S5 |
| G8Q051 | 50S ribosomal protein L18 |
| G8Q053 | RpsH |
| G8Q054 | 30S ribosomal protein S14 |
| G8Q055 | 50S ribosomal protein L5 |
| G8Q057 | 50S ribosomal protein L14 |
| G8Q058 | 30S ribosomal protein S17 |
| G8Q059 | 50S ribosomal protein L29 |
| G8Q060 | 50S ribosomal protein L16 |
| G8Q061 | 30S ribosomal protein S3 |
| G8Q064 | 50S ribosomal protein L2 |
| G8Q065 | 50S ribosomal protein L23 |
| G8Q066 | 50S ribosomal protein L4 |
| G8Q067 | 50S ribosomal protein L3 |
| G8Q068 | 30S ribosomal protein S10 |
| G8Q069 | Elongation factor Tu |
| G8Q070 | Elongation factor G |
| G8Q071 | 30S ribosomal protein S7 |
| G8Q072 | 30S ribosomal protein S12 |
| G8Q073 | DNA-directed RNA polymerase subunit beta |
| G8Q074 | DNA-directed RNA polymerase subunit beta |
| G8Q076 | 50S ribosomal protein L10 |
| G8Q077 | 50S ribosomal protein L1 |
| G8Q078 | 50S ribosomal protein L11 |
| G8Q081 | Elongation factor Tu |
| G8Q0C0 | SerA |
| G8Q0Z6 | Acyl-CoA synthetase(AMP-forming)/AMP-acid ligase |
| G8Q101 | Butyryl-CoA dehydrogenase |
| G8Q151 | EtfA |
| G8Q152 | EtfB |
| G8Q183 | FabG |
| G8Q186 | 50S ribosomal protein L32 |
| G8Q194 | PrkA |
| G8Q1A2 | 30S ribosomal protein S21 |
| G8Q1A4 | RNA polymerase sigma factor RpoD |
| G8Q1B3 | Lysine 2-monooxygenase |
| G8Q1C3 | Acyl-CoA dehydrogenase |
| G8Q1C6 | Acyl-CoA dehydrogenase |
| G8Q1K1 | TypA/BipA |
| G8Q1M7 | Glucans biosynthesis protein G |
| G8Q1R2 | DNA gyrase subunit A |
| G8Q1R4 | PheA |
| G8Q1R8 | 30S ribosomal protein S1 |
| G8Q1W2 | Oprl |
| G8Q1W4 | Phospho-2-dehydro-3-deoxyheptonate aldolase |
| G8Q1W8 | CysB |
| G8Q1X2 | Elongation factor P |
| G8Q262 | Ribonuclease E |
| G8Q2D2 | Chaperone protein HtpG |
| G8Q2G8 | NAD/NADP-dependent betaine aldehyde dehydrogenase |
| G8Q2I4 | Fructose-1,6-bisphosphate aldolase |
| G8Q2J1 | S-adenosylmethionine synthase |
| G8Q2K3 | Adenosylhomocysteinase |
| G8Q2K7 | ATP-dependent RNA helicase RhlE |
| G8Q2L8 | ATP-dependent protease ATPase subunit HslU |
| G8Q2N5 | GltB |
| G8Q2V1 | ATP-dependent RNA helicase DeaD |
| G8Q2W0 | Putative ATP/GTP-binding protein |
| G8Q2Z3 | Siderophore biosynthesis protein |
| G8Q2Z5 | PvdL |
| G8Q302 | PvdH |
| G8Q307 | Phosphoenolpyruvate synthase |
| G8Q314 | OprF |
| G8Q3B1 | Succinate--CoA ligase [ADP-forming] subunit alpha |
| G8Q3B2 | Succinate--CoA ligase [ADP-forming] subunit beta |
| G8Q3B3 | Dihydrolipoyl dehydrogenase |
| G8Q3B4 | Dihydrolipoyllysine-residue succinyltransferase component of 2-oxoglutarate dehydrogenase complex |
| G8Q3B5 | SucA |
| G8Q3B7 | Succinate dehydrogenase flavoprotein subunit |
| G8Q3L1 | Aspartate carbamoyltransferase |
| G8Q3L2 | PyrC |
| G8Q3P4 | Phosphopantetheine adenylyltransferase |
| G8Q3Q0 | Dihydroxy-acid dehydratase |
| G8Q3S2 | Bifunctional protein PutA |
| G8Q3T1 | Acetyltransferase component of pyruvate dehydrogenase complex |
| G8Q3T2 | Pyruvate dehydrogenase E1 component |
| G8Q3V3 | Bifunctional protein HldE |
| G8Q3X1 | DNA topoisomerase 4 subunit B |
| G8Q3X4 | DNA topoisomerase 4 subunit A |
| G8Q3X7 | Phosphoserine phosphatase |
| G8Q451 | PvdD |
| G8Q452 | PvdJ1 |
| G8Q453 | PvdJ2 |
| G8Q454 | Non-ribosomal peptide synthetase modules, amino acid adenylation, pyoverdine |
| G8Q4K7 | Peptidyl-prolyl cis-trans isomerase |
| G8Q4L0 | Recombination-associated protein RdgC |
| G8Q4R4 | SpuB |
| G8Q4R6 | SpuI |
| G8Q4T4 | Amino-acid acetyltransferase |
| G8Q4W1 | Ribonuclease R |
| G8Q4W2 | 23S rRNA (guanosine-2-O)-methyltransferase RlmB |
| G8Q4W3 | 30S ribosomal protein S6 |
| G8Q4W6 | 50S ribosomal protein L9 |
| G8Q4Y7 | Short-chain dehydrogenase reductase sdr |
| G8Q503 | CbpA |
| G8Q539 | PvdA |
| G8Q568 | Aminotransferase |
| G8Q575 | 16 kDa heat shock protein A |
| G8Q587 | Acetyl-coenzyme A carboxylase carboxyl transferase subunit beta |
| G8Q591 | Amidophosphoribosyltransferase |
| G8Q5K8 | Corrinoid adenosyltransferase |
| G8Q5L6 | Phosphoribosylformylglycinamidine cyclo-ligase |
| G8Q5M3 | RelA |
| G8Q5P8 | Ribonucleoside-diphosphate reductase |
| G8Q5R9 | AmrZ |
| G8Q5S8 | Transcription termination factor Rho |
| G8Q5T5 | Ppx |
| G8Q5T6 | Polyphosphate kinase |
| G8Q609 | Biotin carboxylase |
| G8Q617 | Biosynthetic arginine decarboxylase |
| G8Q6F4 | Threonine--tRNA ligase |
| G8Q6F5 | Translation initiation factor IF-3 |
| G8Q6F7 | 50S ribosomal protein L20 |
| G8Q6F8 | Phenylalanine--tRNA ligase alpha subunit |
| G8Q6H7 | AtoB |
| G8Q6P2 | Aspartokinase |
| G8Q6P3 | Alanine--tRNA ligase |
| G8Q6R1 | Ribonucleoside-diphosphate reductase subunit beta |
| G8Q6W0 | 60 kDa chaperonin |
| G8Q6W1 | 10 kDa chaperonin |
| G8Q6Y1 | Acetylglutamate kinase |
| G8Q6Z8 | OxyR |
| G8Q724 | ClpB |
| G8Q7C8 | Phenylalanine--tRNA ligase beta subunit |
| G8Q7V1 | Alkyl hydroperoxide reductase subunit C-like protein |
| G8Q7W4 | Glycerol-3-phosphate dehydrogenase |
| G8Q7X8 | SdaA |
| G8Q7Z3 | Tol-Pal system protein TolB |
| G8Q802 | Aspartate--tRNA(Asp/Asn) ligase |
| G8Q839 | AccC |
| G8Q861 | Cyclopropane-fatty-acyl-phospholipid synthase |
| G8Q893 | Glutamine--fructose-6-phosphate aminotransferase [isomerizing] |
| G8Q895 | Bifunctional protein GlmU |
| G8Q897 | ATP synthase subunit beta |
| G8Q8E0 | Chaperone protein DnaK |
| G8Q8E1 | Chaperone protein DnaJ |
| G8Q8E3 | Carbamoyl-phosphate synthase small chain |
| G8Q8E4 | Carbamoyl-phosphate synthase large chain |
| G8Q8F1 | Phosphoglucosamine mutase |
| G8Q8F4 | Transcription termination/antitermination protein NusA |
| G8Q8F5 | Translation initiation factor IF-2 |
| G8Q8F9 | Polyribonucleotide nucleotidyltransferase |
| G8Q8M6 | NADH-quinone oxidoreductase |
| G8Q8M7 | NADH-quinone oxidoreductase subunit F |
| G8Q8M9 | NADH-quinone oxidoreductase subunit C/D |
| G8Q8P2 | Isocitrate dehydrogenase [NADP] |
| G8Q8P5 | ClpA |
| G8Q8Q4 | Serine--tRNA ligase |
| G8Q8Y7 | Protein translocase subunit SecA |
| G8Q8Z5 | MvaT |
| G8Q906 | Pyruvate kinase |
| G8Q963 | Phosphoribosylformylglycinamidine synthase |
| G8Q967 | ATP synthase gamma chain |
| G8Q968 | ATP synthase subunit alpha |
| G8Q970 | ATP synthase subunit b |
| G8Q985 | Peptide chain release factor 3 |
| G8Q9B3 | MreB |
| G8Q9C0 | TldD |
| G8Q9E4 | UDP-N-acetylglucosamine 1-carboxyvinyltransferase |
| G8Q9X9 | Probable cytosol aminopeptidase |
| G8Q9Y2 | Valine--tRNA ligase |
| G8Q9Y4 | DNA-binding protein HU, form N |
| G8Q9Z8 | ATP-dependent RNA helicase RhlB |
| G8QA06 | PhoH |
| G8QA09 | GDP-mannose 6-dehydrogenase |
| G8QA35 | Cell division protein FtsZ |
| G8QA36 | Cell division protein FtsA |
| G8QA38 | D-alanine--D-alanine ligase |
| G8QA89 | Inositol-1-monophosphatase |
| G8QA93 | Cysteine desulfurase IscS |
| G8QAA4 | 4-hydroxy-3-methylbut-2-en-1-yl diphosphate synthase (flavodoxin) |
| G8QAA5 | Histidine--tRNA ligase |
| G8QAA8 | GTPase Der |
| G8QAB7 | Inosine-5-monophosphate dehydrogenase |
| G8QAI9 | MvaU |
| G8QAL3 | Branched-chain-amino-acid aminotransferase |
| G8QAV2 | 30S ribosomal protein S9 |
| G8QAV3 | 50S ribosomal protein L13 |
| G8QB27 | Lon protease |
| G8QB35 | 50S ribosomal protein L19 |
| G8QB38 | Homoserine dehydrogenase |
| G8QB66 | Lysine--tRNA ligase |
| G8QB98 | 2,3,4,5-tetrahydropyridine-2,6-dicarboxylate N-succinyltransferase |
| G8QB99 | Uncharacterized protein |
| G8QBA8 | Dihydrolipoamide acetyltransferase component of pyruvate dehydrogenase complex |
| G8QBT1 | Phosphate acetyltransferase |
| G8QBT3 | Multifunctional fusion protein |
| G8QBT4 | Sulfate adenylyltransferase subunit 2 |
| G8QBU7 | Probable malate:quinone oxidoreductase |
| G8QBX2 | Ribose-phosphate pyrophosphokinase |
| G8QBX3 | 50S ribosomal protein L25 |
| G8QBZ7 | 30S ribosomal protein S2 |
| G8QBZ8 | Elongation factor Ts |
| G8QC06 | Outer membrane chaperone skp |
| G8QC13 | Acetyl-coenzyme A carboxylase carboxyl transferase subunit alpha |
| G8QC15 | CTP synthase |
| G8QC16 | 2-dehydro-3-deoxyphosphooctonate aldolase |
| G8QC56 | Protein RecA |
| G8QC58 | Decarboxylase family protein |
| G8QC75 | Aconitate hydratase B |
| G8QCG4 | GdhB |
| G8QCN6 | Chromosome segregation ATPase |
| G8QCP5 | Acetolactate synthase |
| G8QCR4 | CbrB |
| G8QCU1 | Chaperone protein ClpB |
| **Carboxylate-NPs** | |
| **UNIPROT Code** | **Protein name** |
| G8PW43 | Chromosomal replication initiator protein DnaA |
| G8PW44 | Beta sliding clamp |
| G8PW46 | DNA gyrase subunit B |
| G8PW52 | Glycine-tRNA ligase beta subunit |
| G8PW57 | Proline-tRNA ligase |
| G8PWA9 | Carbamate kinase |
| G8PWB1 | Arginine deiminase |
| G8PWB6 | HemO |
| G8PWB7 | TonB-dependent hemin, ferrichrome receptor |
| G8PWD2 | HexR |
| G8PWF1 | Diaminobutyrate--2-oxoglutarate aminotransferase |
| G8PWW0 | Glutamate 5-kinase |
| G8PWW1 | GTPase Obg |
| G8PWX6 | ABC transporter, ATP-binding protein |
| G8PX08 | Transcriptional regulator, TetR family |
| G8PX53 | Probable GTP-binding protein EngB |
| G8PXD9 | Iron-regulated protein A |
| G8PXI9 | Alcohol dehydrogenase II |
| G8PXS2 | LiuR |
| G8PXU5 | Probable transcriptional regulatory protein PSF113_3903 |
| G8PXX5 | Glutamate-1-semialdehyde 2,1-aminomutase |
| G8PXX9 | Phosphate starvation-inducible ATPase PhoH with RNA binding motif |
| G8PXY1 | CorC |
| G8PY19 | Hpa2 |
| G8PY94 | GcdH |
| G8PYE9 | Periplasmic serine endoprotease DegP-like |
| G8PYF8 | Phosphoribosylaminoimidazole-succinocarboxamide synthase |
| G8PYK4 | Thiol peroxidase |
| G8PYR7 | DNA-binding protein HU-beta |
| G8PYR8 | Lon protease |
| G8PYR9 | ATP-dependent Clp protease ATP-binding subunit ClpX |
| G8PYS1 | Trigger factor |
| G8PYT2 | Uncharacterized protein |
| G8PYV7 | StaS |
| G8PYX4 | Glyceraldehyde-3-phosphate dehydrogenase |
| G8PYZ0 | Universal stress protein |
| G8PZ16 | Uncharacterized protein |
| G8PZ43 | YbbN |
| G8PZ64 | Single-stranded DNA-binding protein |
| G8PZ66 | UvrABC system protein A |
| G8PZ69 | 50S ribosomal protein L17 |
| G8PZ70 | DNA-directed RNA polymerase subunit alpha |
| G8PZ71 | 30S ribosomal protein S4 |
| G8PZ72 | 30S ribosomal protein S11 |
| G8PZ73 | 30S ribosomal protein S13 |
| G8PZE7 | PtsP |
| G8PZI4 | SrmB |
| G8PZM4 | Aspartate aminotransferase/Aromatic-amino-acid aminotransferase |
| G8PZM5 | Putative pterin-4-alpha-carbinolamine dehydratase |
| G8PZQ4 | KaiC |
| G8PZW2 | Glyceraldehyde-3-phosphate dehydrogenase, putative |
| G8PZZ2 | Nucleoid-associated protein PSF113_4021 |
| G8PZZ3 | DNA polymerase III subunit gamma/tau |
| G8Q000 | Transcriptional regulator, GntR family |
| G8Q005 | Transcriptional regulator, GntR family |
| G8Q010 | Ribosomal RNA large subunit methyltransferase K/L |
| G8Q038 | Oxidoreductase, short chain dehydrogenase/reductase family |
| G8Q047 | Protein translocase subunit SecY |
| G8Q048 | 50S ribosomal protein L15 |
| G8Q050 | 30S ribosomal protein S5 |
| G8Q051 | 50S ribosomal protein L18 |
| G8Q052 | RplF |
| G8Q053 | RpsH |
| G8Q054 | 30S ribosomal protein S14 |
| G8Q055 | 50S ribosomal protein L5 |
| G8Q056 | 50S ribosomal protein L24 |
| G8Q057 | 50S ribosomal protein L14 |
| G8Q058 | 30S ribosomal protein S17 |
| G8Q059 | 50S ribosomal protein L29 |
| G8Q060 | 50S ribosomal protein L16 |
| G8Q061 | 30S ribosomal protein S3 |
| G8Q063 | 30S ribosomal protein S19 |
| G8Q064 | 50S ribosomal protein L2 |
| G8Q065 | 50S ribosomal protein L23 |
| G8Q066 | 50S ribosomal protein L4 |
| G8Q067 | 50S ribosomal protein L3 |
| G8Q068 | 30S ribosomal protein S10 |
| G8Q069 | Elongation factor Tu |
| G8Q070 | Elongation factor G |
| G8Q071 | 30S ribosomal protein S7 |
| G8Q072 | 30S ribosomal protein S12 |
| G8Q073 | DNA-directed RNA polymerase subunit beta |
| G8Q074 | DNA-directed RNA polymerase subunit beta |
| G8Q075 | 50S ribosomal protein L7/L12 |
| G8Q076 | 50S ribosomal protein L10 |
| G8Q077 | 50S ribosomal protein L1 |
| G8Q078 | 50S ribosomal protein L11 |
| G8Q079 | Transcription termination/antitermination protein NusG |
| G8Q081 | Elongation factor Tu |
| G8Q085 | Tyrosine--tRNA ligase |
| G8Q0C0 | SerA |
| G8Q0E6 | AmgR |
| G8Q151 | EtfA |
| G8Q152 | EtfB |
| G8Q174 | Transcriptional regulator, TetR family |
| G8Q182 | Acyl carrier protein |
| G8Q183 | FabG |
| G8Q186 | 50S ribosomal protein L32 |
| G8Q194 | PrkA |
| G8Q197 | Multifunctional CCA protein |
| G8Q1A2 | 30S ribosomal protein S21 |
| G8Q1A4 | RNA polymerase sigma factor RpoD |
| G8Q1C6 | Acyl-CoA dehydrogenase |
| G8Q1K1 | TypA/BipA |
| G8Q1P0 | Ubiquinone/menaquinone biosynthesis C-methyltransferase UbiE |
| G8Q1P3 | PhaF |
| G8Q1P9 | Uncharacterized protein |
| G8Q1R2 | DNA gyrase subunit A |
| G8Q1R8 | 30S ribosomal protein S1 |
| G8Q1W2 | Oprl |
| G8Q1W4 | Phospho-2-dehydro-3-deoxyheptonate aldolase |
| G8Q1X2 | Elongation factor P |
| G8Q262 | Ribonuclease E |
| G8Q2C7 | KasI/FabB |
| G8Q2D2 | Chaperone protein HtpG |
| G8Q2I4 | Fructose-1,6-bisphosphate aldolase |
| G8Q2J1 | S-adenosylmethionine synthase |
| G8Q2K3 | Adenosylhomocysteinase |
| G8Q2K7 | ATP-dependent RNA helicase RhlE |
| G8Q2K8 | UPF0312 protein PSF113_5506 |
| G8Q2L0 | Amine oxidase [flavin-containing] A |
| G8Q2L8 | ATP-dependent protease ATPase subunit HslU |
| G8Q2V1 | ATP-dependent RNA helicase DeaD |
| G8Q2Z3 | Siderophore biosynthesis protein |
| G8Q2Z5 | PvdL |
| G8Q302 | PvdH |
| G8Q307 | Phosphoenolpyruvate synthase |
| G8Q314 | OprF |
| G8Q3B1 | Succinate--CoA ligase [ADP-forming] subunit alpha |
| G8Q3B2 | Succinate--CoA ligase [ADP-forming] subunit beta |
| G8Q3B3 | Dihydrolipoyl dehydrogenase |
| G8Q3B4 | Dihydrolipoyllysine-residue succinyltransferase component of 2-oxoglutarate dehydrogenase complex |
| G8Q3B5 | SucA |
| G8Q3B6 | SdhB |
| G8Q3B7 | Succinate dehydrogenase flavoprotein subunit |
| G8Q3P4 | Phosphopantetheine adenylyltransferase |
| G8Q3P9 | RlmI |
| G8Q3S2 | Bifunctional protein PutA |
| G8Q3S8 | Peptide methionine sulfoxide reductase MsrA |
| G8Q3T1 | Acetyltransferase component of pyruvate dehydrogenase complex |
| G8Q3T2 | Pyruvate dehydrogenase E1 component |
| G8Q3X1 | DNA topoisomerase 4 subunit B |
| G8Q3X4 | DNA topoisomerase 4 subunit A |
| G8Q3Y1 | Signal transduction protein |
| G8Q453 | PvdJ2 |
| G8Q454 | Non-ribosomal peptide synthetase modules, amino acid adenylation, pyoverdine |
| G8Q4F2 | Universal stress protein family 5 |
| G8Q4I4 | Site-determining protein |
| G8Q4I5 | Cell division topological specificity factor |
| G8Q4K7 | Peptidyl-prolyl cis-trans isomerase |
| G8Q4L0 | Recombination-associated protein RdgC |
| G8Q4R4 | SpuB |
| G8Q4T5 | Acetylornithine deacetylase |
| G8Q4W1 | Ribonuclease R |
| G8Q4W2 | 23S rRNA (guanosine-2-O-)-methyltransferase RlmB |
| G8Q4W3 | 30S ribosomal protein S6 |
| G8Q4W4 | 30S ribosomal protein S18 |
| G8Q4W6 | 50S ribosomal protein L9 |
| G8Q4Y7 | Short-chain dehydrogenase reductase sdr |
| G8Q503 | CbpA |
| G8Q539 | PvdA |
| G8Q568 | Aminotransferase |
| G8Q575 | 16 kDa heat shock protein A |
| G8Q587 | Acetyl-coenzyme A carboxylase carboxyl transferase subunit beta |
| G8Q591 | Amidophosphoribosyltransferase |
| G8Q5K8 | Corrinoid adenosyltransferase |
| G8Q5L6 | Phosphoribosylformylglycinamidine cyclo-ligase |
| G8Q5M1 | Nucleoprotein/polynucleotide-associated enzyme |
| G8Q5M3 | RelA |
| G8Q5P8 | Ribonucleoside-diphosphate reductase |
| G8Q5R9 | AmrZ |
| G8Q5S8 | Transcription termination factor Rho |
| G8Q5S9 | Thioredoxin |
| G8Q5T5 | Ppx |
| G8Q5T6 | Polyphosphate kinase |
| G8Q5U9 | Glutathione-regulated potassium-efflux system ATP-binding protein |
| G8Q5V1 | AlgP |
| G8Q5V8 | AlgR |
| G8Q5Z9 | D-amino acid dehydrogenase |
| G8Q609 | Biotin carboxylase |
| G8Q6F2 | CspG |
| G8Q6F4 | Threonine--tRNA ligase |
| G8Q6F5 | Translation initiation factor IF-3 |
| G8Q6F7 | 50S ribosomal protein L20 |
| G8Q6F8 | Phenylalanine--tRNA ligase alpha subunit |
| G8Q6H7 | AtoB |
| G8Q6I2 | General stress protein |
| G8Q6P2 | Aspartokinase |
| G8Q6P3 | Alanine--tRNA ligase |
| G8Q6R1 | Ribonucleoside-diphosphate reductase subunit beta |
| G8Q6W0 | 60 kDa chaperonin |
| G8Q6W1 | 10 kDa chaperonin |
| G8Q6X4 | 50S ribosomal protein L33 |
| G8Q6X5 | RpmB |
| G8Q6Y0 | AlgC |
| G8Q6Y1 | Acetylglutamate kinase |
| G8Q6Z8 | OxyR |
| G8Q7C8 | Phenylalanine--tRNA ligase beta subunit |
| G8Q7L7 | Peptidylprolyl isomerase |
| G8Q7U7 | Argininosuccinate synthase |
| G8Q7V1 | Alkyl hydroperoxide reductase subunit C-like protein |
| G8Q7V4 | Glutaredoxin |
| G8Q7W0 | Cys-tRNA(Pro)/Cys-tRNA(Cys) deacylase |
| G8Q7W4 | Glycerol-3-phosphate dehydrogenase |
| G8Q7X8 | SdaA |
| G8Q7Y0 | CspA |
| G8Q802 | Aspartate--tRNA(Asp/Asn) ligase |
| G8Q805 | Non-specific DNA-binding protein Dps / Iron-binding ferritin-like antioxidant protein / Ferroxidase |
| G8Q812 | UPF0234 protein PSF113_4629 |
| G8Q845 | Putative membrane protein |
| G8Q861 | Cyclopropane-fatty-acyl-phospholipid synthase |
| G8Q895 | Bifunctional protein GlmU |
| G8Q896 | ATP synthase epsilon chain |
| G8Q897 | ATP synthase subunit beta |
| G8Q8D7 | Ferric uptake regulation protein |
| G8Q8D9 | Protein GrpE |
| G8Q8E0 | Chaperone protein DnaK |
| G8Q8E1 | Chaperone protein DnaJ |
| G8Q8E2 | 4-hydroxy-tetrahydrodipicolinate reductase |
| G8Q8E3 | Carbamoyl-phosphate synthase small chain |
| G8Q8E4 | Carbamoyl-phosphate synthase large chain |
| G8Q8E5 | Transcription elongation factor GreA |
| G8Q8F1 | Phosphoglucosamine mutase |
| G8Q8F4 | Transcription termination/antitermination protein NusA |
| G8Q8F5 | Translation initiation factor IF-2 |
| G8Q8F6 | Ribosome-binding factor A |
| G8Q8H4 | SpeC |
| G8Q8M9 | NADH-quinone oxidoreductase subunit C/D |
| G8Q8P2 | Isocitrate dehydrogenase [NADP] |
| G8Q8P5 | ClpA |
| G8Q8Q4 | Serine--tRNA ligase |
| G8Q8Y7 | Protein translocase subunit SecA |
| G8Q8Z5 | MvaT |
| G8Q906 | Pyruvate kinase |
| G8Q963 | Phosphoribosylformylglycinamidine synthase |
| G8Q967 | ATP synthase gamma chain |
| G8Q968 | ATP synthase subunit alpha |
| G8Q969 | ATP synthase subunit delta |
| G8Q970 | ATP synthase subunit b |
| G8Q974 | ParB |
| G8Q9B1 | Glutamyl-tRNA(Gln) amidotransferase subunit A |
| G8Q9B3 | MreB |
| G8Q9C1 | UPF0307 protein PSF113_0931 |
| G8Q9D1 | YhbH |
| G8Q9E4 | UDP-N-acetylglucosamine 1-carboxyvinyltransferase |
| G8Q9E5 | ATP phosphoribosyltransferase |
| G8Q9X6 | Elongation factor 4 |
| G8Q9X9 | Probable cytosol aminopeptidase |
| G8Q9Y4 | DNA-binding protein HU, form N |
| G8QA09 | GDP-mannose 6-dehydrogenase |
| G8QA19 | AlgF |
| G8QA35 | Cell division protein FtsZ |
| G8QA38 | D-alanine--D-alanine ligase |
| G8QA39 | UDP-N-acetylmuramate--L-alanine ligase |
| G8QA48 | Ribosomal RNA small subunit methyltransferase H |
| G8QA92 | IscR |
| G8QAA0 | Nucleoside diphosphate kinase |
| G8QAA4 | 4-hydroxy-3-methylbut-2-en-1-yl diphosphate synthase (flavodoxin) |
| G8QAA5 | Histidine--tRNA ligase |
| G8QAA8 | GTPase Der |
| G8QAB7 | Inosine-5-monophosphate dehydrogenase |
| G8QAG6 | Decarboxylase family protein |
| G8QAI9 | MvaU |
| G8QAJ3 | 4-hydroxyphenylpyruvate dioxygenase |
| G8QAM8 | 3-oxoacyl-[acyl-carrier protein] reductase |
| G8QAV2 | 30S ribosomal protein S9 |
| G8QAV3 | 50S ribosomal protein L13 |
| G8QB27 | Lon protease |
| G8QB32 | 30S ribosomal protein S16 |
| G8QB35 | 50S ribosomal protein L19 |
| G8QB38 | Homoserine dehydrogenase |
| G8QB66 | Lysine--tRNA ligase |
| G8QB72 | Outer membrane lipoprotein omp16 |
| G8QB87 | Putative exported protein |
| G8QB98 | 2,3,4,5-tetrahydropyridine-2,6-dicarboxylate N-succinyltransferase |
| G8QB99 | Uncharacterized protein |
| G8QBA6 | Methionine aminopeptidase |
| G8QBA8 | Dihydrolipoamide acetyltransferase component of pyruvate dehydrogenase complex |
| G8QBL5 | Enoyl-[acyl-carrier-protein] reductase [NADH] |
| G8QBM9 | Nadph-dependent fmn reductase |
| G8QBU7 | Probable malate:quinone oxidoreductase |
| G8QBX2 | Ribose-phosphate pyrophosphokinase |
| G8QBX3 | 50S ribosomal protein L25 |
| G8QBZ7 | 30S ribosomal protein S2 |
| G8QBZ8 | Elongation factor Ts |
| G8QC00 | Ribosome-recycling factor |
| G8QC01 | Ditrans,polycis-undecaprenyl-diphosphate synthase ((2E,6E)-farnesyl-diphosphate specific) |
| G8QC06 | Outer membrane chaperone skp |
| G8QC13 | Acetyl-coenzyme A carboxylase carboxyl transferase subunit alpha |
| G8QC15 | CTP synthase |
| G8QC16 | 2-dehydro-3-deoxyphosphooctonate aldolase |
| G8QC17 | Enolase |
| G8QC21 | S-(Hydroxymethyl)glutathione dehydrogenase |
| G8QC31 | DNA mismatch repair protein MutS |
| G8QC56 | Protein RecA |
| G8QC75 | Aconitate hydratase B |
| G8QCG4 | GdhB |
| G8QCP5 | Acetolactate synthase |
| G8QCQ0 | Uncharacterized protein |
| G8QCR4 | CbrB |
| G8QCR5 | Poly(A) polymerase I |
| G8QCS5 | Osmotically inducible protein Y |
| G8QCU1 | Chaperone protein ClpB |
| **Aromatic-NPs** | |
| **UNIPROT Code** | **Protein name** |
| G8Q802 | Aspartate-tRNA(Asp/Asn) ligase |
| G8PW44 | Beta sliding clamp |
| G8PWB1 | Arginine deiminase |
| G8PWB6 | HemO |
| G8PWD4 | 6-phosphogluconolactonase |
| G8PWW1 | GTPase Obg |
| G8PWX6 | ABC transporter, ATP-binding protein |
| G8PXI9 | Alcohol dehydrogenase II |
| G8PXU5 | Probable transcriptional regulatory protein PSF113_3903 |
| G8PYF8 | Phosphoribosylaminoimidazole-succinocarboxamide synthase |
| G8PYR8 | Lon protease |
| G8PYR9 | ATP-dependent Clp protease ATP-binding subunit ClpX |
| G8PYS1 | Trigger factor |
| G8PYX4 | Glyceraldehyde-3-phosphate dehydrogenase |
| G8PZ64 | Single-stranded DNA-binding protein |
| G8PZ70 | DNA-directed RNA polymerase subunit alpha |
| G8PZ71 | 30S ribosomal protein S4 |
| G8PZ72 | 30S ribosomal protein S11 |
| G8PZ73 | 30S ribosomal protein S13 |
| G8PZL0 | Yeca family protein |
| G8PZM5 | Putative pterin-4-alpha-carbinolamine dehydratase |
| G8Q048 | 50S ribosomal protein L15 |
| G8Q050 | 30S ribosomal protein S5 |
| G8Q051 | 50S ribosomal protein L18 |
| G8Q052 | RplF |
| G8Q053 | RpsH |
| G8Q054 | 30S ribosomal protein S14 |
| G8Q061 | 30S ribosomal protein S3 |
| G8Q063 | 30S ribosomal protein S19 |
| G8Q064 | 50S ribosomal protein L2 |
| G8Q067 | 50S ribosomal protein L3 |
| G8Q068 | 30S ribosomal protein S10 |
| G8Q069 | Elongation factor Tu |
| G8Q070 | Elongation factor G |
| G8Q071 | 30S ribosomal protein S7 |
| G8Q072 | 30S ribosomal protein S12 |
| G8Q073 | DNA-directed RNA polymerase subunit beta |
| G8Q074 | DNA-directed RNA polymerase subunit beta |
| G8Q076 | 50S ribosomal protein L10 |
| G8Q077 | 50S ribosomal protein L1 |
| G8Q078 | 50S ribosomal protein L11 |
| G8Q081 | Elongation factor Tu |
| G8Q0C0 | SerA |
| G8Q151 | EtfA |
| G8Q152 | EtfB |
| G8Q182 | Acyl carrier protein |
| G8Q194 | PrkA |
| G8Q1A2 | 30S ribosomal protein S21 |
| G8Q1R8 | 30S ribosomal protein S1 |
| G8Q1W2 | Oprl |
| G8Q1X2 | Elongation factor P |
| G8Q2D2 | Chaperone protein HtpG |
| G8Q2I4 | Fructose-1,6-bisphosphate aldolase |
| G8Q2L8 | ATP-dependent protease ATPase subunit HslU |
| G8Q2V1 | ATP-dependent RNA helicase DeaD |
| G8Q302 | PvdH |
| G8Q307 | Phosphoenolpyruvate synthase |
| G8Q3B1 | Succinate--CoA ligase [ADP-forming] subunit alpha |
| G8Q3B2 | Succinate--CoA ligase [ADP-forming] subunit beta |
| G8Q3B4 | Dihydrolipoyllysine-residue succinyltransferase component of 2-oxoglutarate dehydrogenase complex |
| G8Q3B5 | SucA |
| G8Q3T2 | Pyruvate dehydrogenase E1 component |
| G8Q4W3 | 30S ribosomal protein S6 |
| G8Q4W4 | 30S ribosomal protein S18 |
| G8Q4W6 | 50S ribosomal protein L9 |
| G8Q5P8 | Ribonucleoside-diphosphate reductase |
| G8Q5R9 | AmrZ |
| G8Q5S8 | Transcription termination factor Rho |
| G8Q5S9 | Thioredoxin |
| G8Q609 | Biotin carboxylase |
| G8Q6D7 | Translational regulator CsrA |
| G8Q6F2 | CspG |
| G8Q6F7 | 50S ribosomal protein L20 |
| G8Q6H7 | AtoB |
| G8Q6R1 | Ribonucleoside-diphosphate reductase subunit beta |
| G8Q6W0 | 60 kDa chaperonin |
| G8Q6W1 | 10 kDa chaperonin |
| G8Q6Y0 | AlgC |
| G8Q6Y2 | Orotate phosphoribosyltransferase |
| G8Q7V1 | Alkyl hydroperoxide reductase subunit C-like protein |
| G8Q7V4 | Glutaredoxin |
| G8Q812 | UPF0234 protein PSF113_4629 |
| G8Q8D9 | Protein GrpE |
| G8Q8E0 | Chaperone protein DnaK |
| G8Q8E2 | 4-hydroxy-tetrahydrodipicolinate reductase |
| G8Q8E4 | Carbamoyl-phosphate synthase large chain |
| G8Q8F4 | Transcription termination/antitermination protein NusA |
| G8Q8F5 | Translation initiation factor IF-2 |
| G8Q8F9 | Polyribonucleotide nucleotidyltransferase |
| G8Q8P2 | Isocitrate dehydrogenase [NADP] |
| G8Q8P5 | ClpA |
| G8Q968 | ATP synthase subunit alpha |
| G8Q9E4 | UDP-N-acetylglucosamine 1-carboxyvinyltransferase |
| G8Q9X9 | Probable cytosol aminopeptidase |
| G8QA35 | Cell division protein FtsZ |
| G8QAA0 | Nucleoside diphosphate kinase |
| G8QAV2 | 30S ribosomal protein S9 |
| G8QB32 | 30S ribosomal protein S16 |
| G8QB35 | 50S ribosomal protein L19 |
| G8QB66 | Lysine--tRNA ligase |
| G8QBX3 | 50S ribosomal protein L25 |
| G8QBZ7 | 30S ribosomal protein S2 |
| G8QBZ8 | Elongation factor Ts |
| G8QC00 | Ribosome-recycling factor |
| G8QC15 | CTP synthase |
| G8QC17 | Enolase |
| G8QC56 | Protein RecA |
| G8QC75 | Aconitate hydratase B |
| G8QCG4 | GdhB |
| G8QCS5 | Osmotically inducible protein Y |
| G8QCU1 | Chaperone protein ClpB |


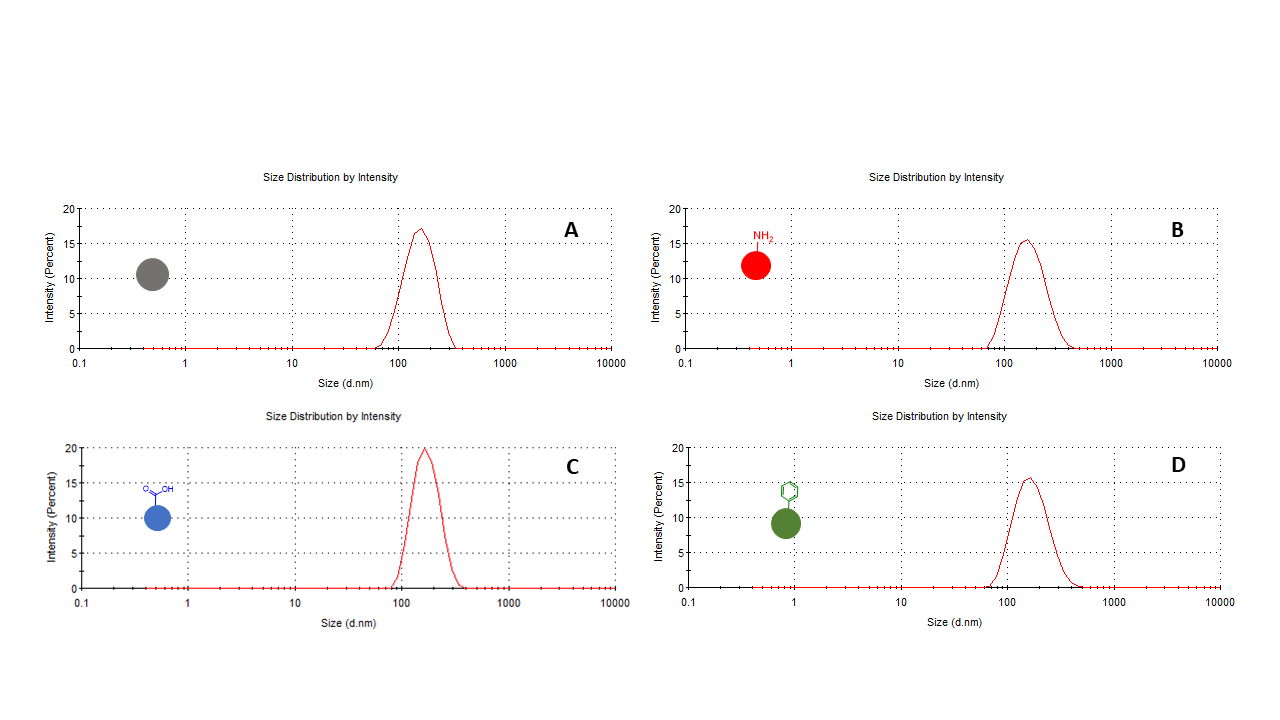


**Figure S1.** Profile of size distribution by intensity using DLS for bare NPs (A), amino-NPs (B), carboxylate-NPS (C) and aromatic-NPs (D).


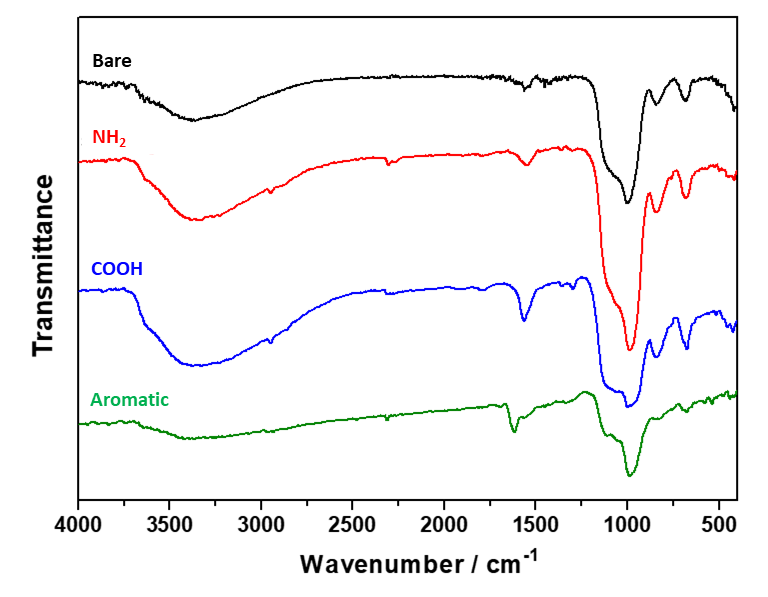


**Figure S2.** FTIR spectra of the synthesised silica nanoparticles.


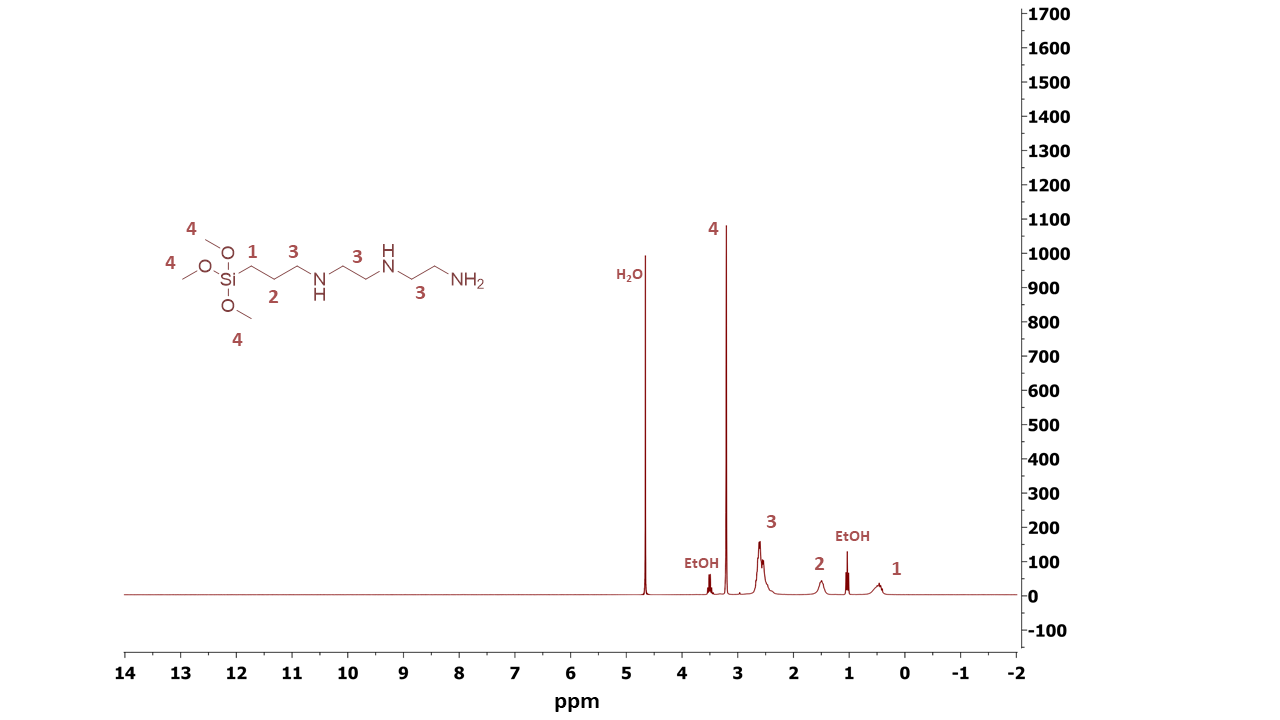


**Figure S3.** ^1^H NMR spectrum of N1-(3-Trimethoxysilylpropyl) diethylenetriamine (DETA) in D_2_O.


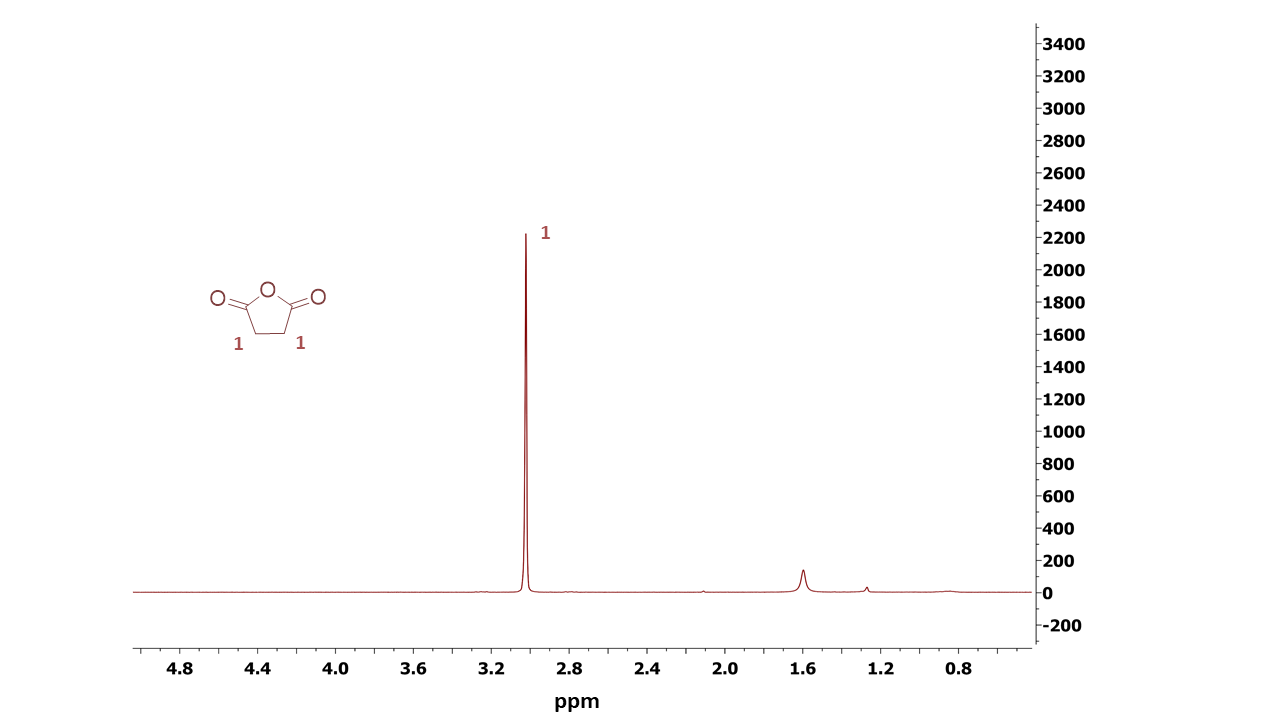


**Figure S4.** ^1^H NMR spectrum of succinic anhydride in CDCl_3_.


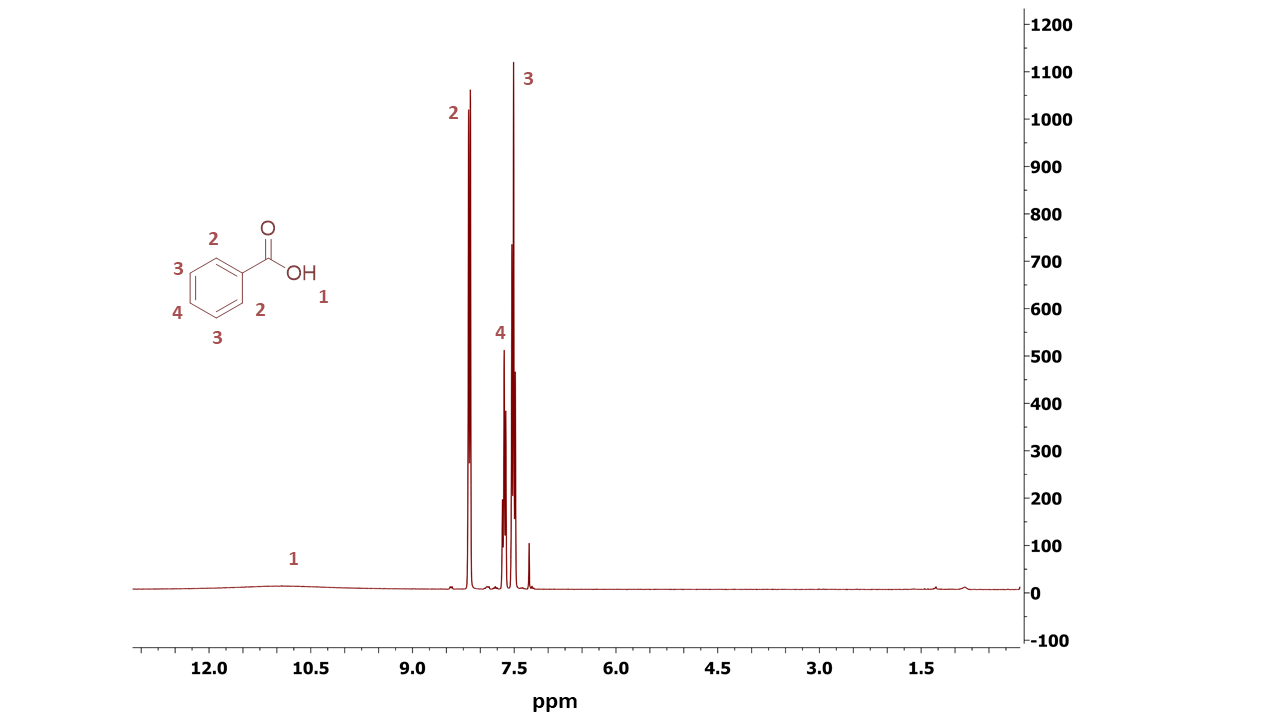


**Figure S5.** ^1^H NMR spectrum of benzoic acid in CDCl_3_.

**
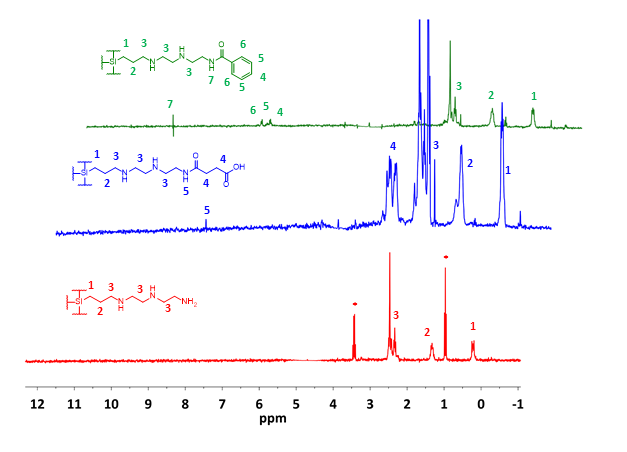
**

**Figure S6.** Dissolution ^1^H NMR spectra of the functionalised silica nanoparticles.

**Figure S7.** Partitioning quotient (amount of Rose-Bengal dye bound to NPs per amount of free dye in solution) as a function of surface area of increasing concentrations of NPs. The steep slope existent only for aromatic-NPs is a clear evidence of increased hydrophobicity.


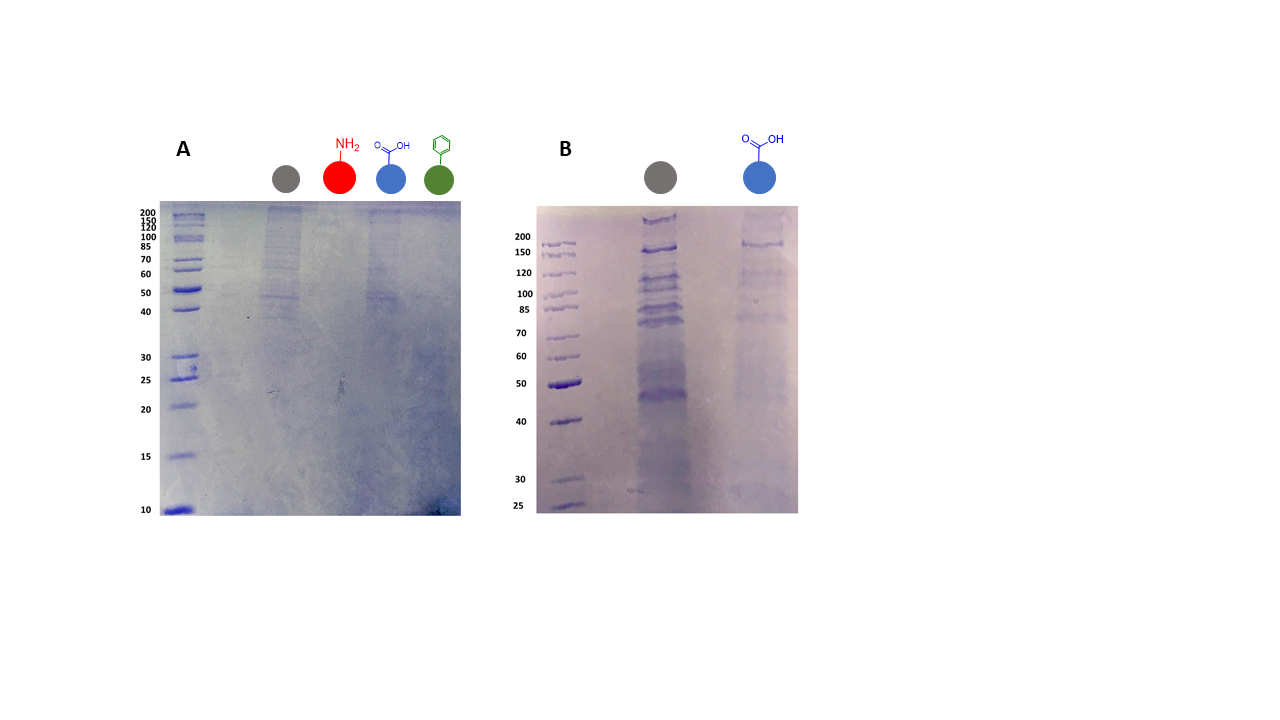


**Figure S8.** SDS-PAGE 10 % of protein corona of the four types of silica nanoparticles (A) and SDS-PAGE 8% of the protein corona of bare SNPs and SNPs-COOH.


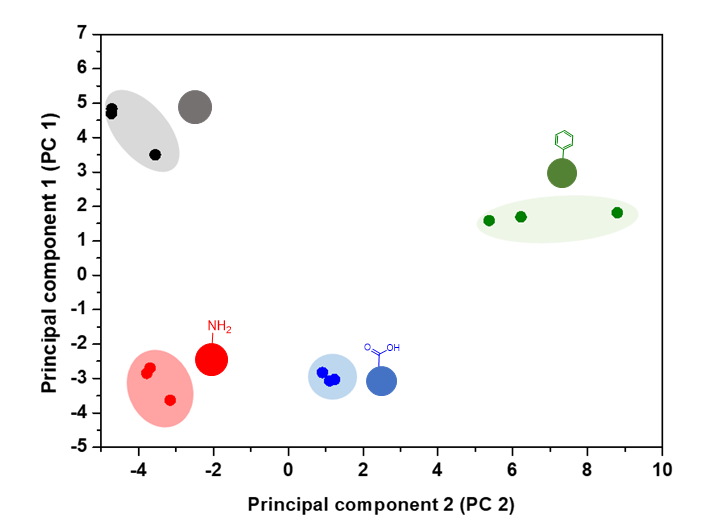


**Figure S9.** Principal Component Analysis of protein coronas after Label-free Quantification (LFQ) proteomics.

**
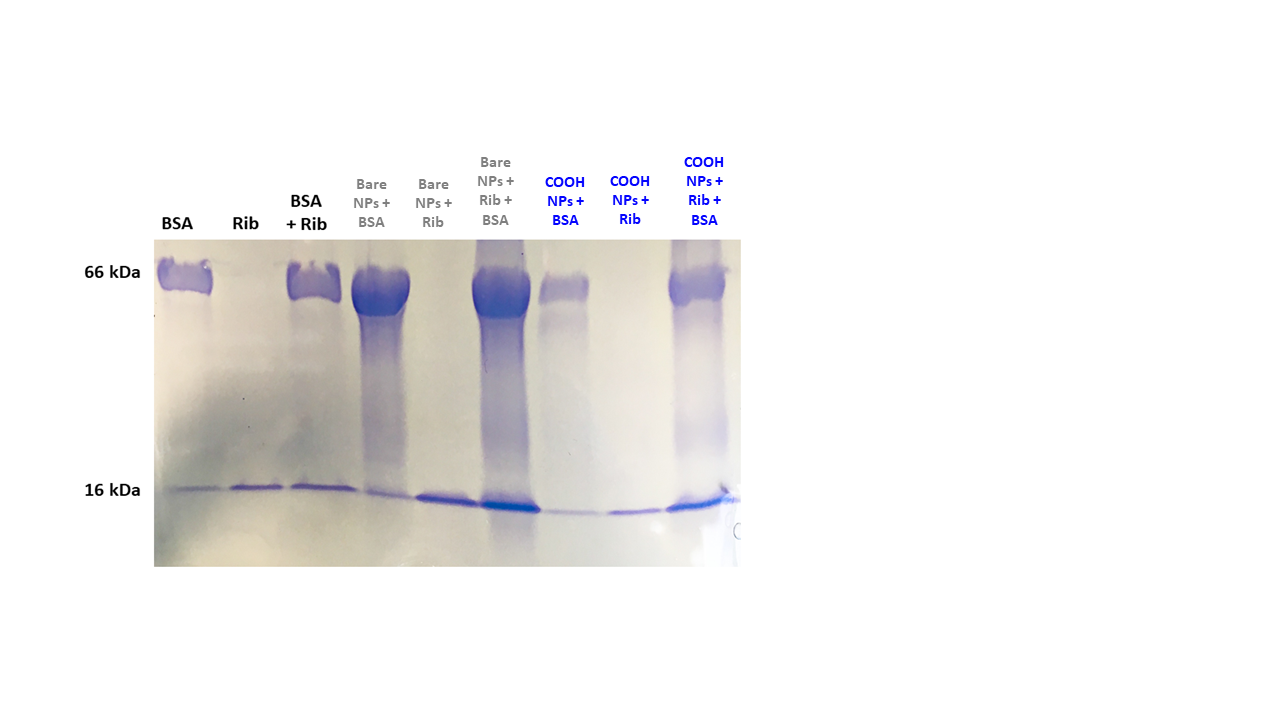
**

**Figure S10.** SDS-PAGE 10 % of BSA and ribonuclease in, respectively, pure form (lanes 1-3), in a mixture (1:1) and as protein coronas of bare SNPs (lanes 4-6) and SNPs-COOH (lanes 7-9).
